# Carryover Effects on Reproduction Can Buffer Against Mortality‐Driven Population Declines at Elevated Developmental Temperatures

**DOI:** 10.1111/ele.70264

**Published:** 2025-11-11

**Authors:** Noah T. Leith, Anthony Macchiano, Brigitte Tenhumberg, Inaya Smith, Jake P. Woods, Kasey D. Fowler‐Finn

**Affiliations:** ^1^ Department of Biology Saint Louis University St. Louis Missouri USA; ^2^ Department of Biological Sciences University of Pittsburgh Pittsburgh Pennsylvania USA; ^3^ Department of Environmental Science, Policy, and Management University of California Berkeley Berkeley California USA; ^4^ Environmental Protection Agency Washington DC USA; ^5^ School of Biological Sciences University of Nebraska‐Lincoln Lincoln Nebraska USA; ^6^ Department of Biological Sciences Harris Stowe State University St. Louis Missouri USA; ^7^ College of Public Health and Social Justice Saint Louis University St. Louis Missouri USA; ^8^ Department of Integrative Biology University of South Florida Tampa Florida USA

**Keywords:** climate change, extinction, fertility, genitalia, mating behaviour, phenotypic plasticity, population dynamics, sex‐specific response, sexual selection, thermal tolerance

## Abstract

Carryover effects broadly influence adult performance and population persistence. Although variation in developmental environments can have contrasting effects on survival and reproduction, current predictive models of vulnerability to environmental change typically ignore simultaneous changes in both fitness components. We tested how developmental temperatures shape survival and multiple aspects of reproduction in a plant‐living insect (*Enchenopa binotata* treehoppers) and used population dynamic models to examine the cumulative impact of these complex effects on population growth. We show that elevated developmental temperatures increase adult fertility, offsetting reduced juvenile survival and potentially forestalling population declines following global warming. Moreover, pairing mates from different developmental temperatures exacerbated morphological mismatches between sexes, reducing mating success and sperm transfer. Our findings thereby provide novel evidence that thermal carryover effects can generate assortative mating and selection on adult reproductive morphology, in addition to shaping the relative importance of survival and reproduction for persistence in warming climates.

## Graphical abstract

We show that hotter juvenile temperatures can increase adult fertility in an emerging model insect system. These reproductive benefits may be crucial for insect populations to avoid extinction during global warming, which often reduces survival rates. Population dynamics estimated from lethal stress alone, or without considering carryover effects from developmental plasticity across animals' different life stages, may be unreliable for predicting extinction risk in response to climate change.

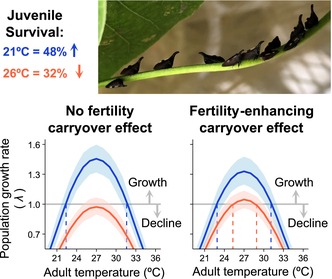

## 1 Introduction

Increasingly extreme and unpredictable temperatures are leading threats to biodiversity (Bellard et al. 2012; Thomas et al. 2004). Understanding the processes shaping extinction risk in the wake of global warming is therefore paramount to forestalling biodiversity loss. Although breakdowns in many fitness components could culminate in extinction, population persistence in warming climates—particularly for short‐lived organisms—may hinge specifically on reproduction (Franco and Silvertown 2004; Le Coeur et al. 2022). Reproduction is often more thermally sensitive than survival, causing the thermal limits of fertility to disproportionately influence species' vulnerability to climate change (Dahlke et al. 2020; van Heerwaarden and Sgrò 2021; Ørsted et al. 2024; Walsh et al. 2019). However, the thermal sensitivity of reproduction depends on the temperatures experienced across the entire life cycle; developmental environments can determine both overall performance and how adults respond to environmental variation later in life (i.e., carryover effects, Box 1; Moore and Martin 2019). Although some carryover effect models imply that adverse developmental environments consistently reduce both survival and reproduction (Lindström 1999; Van De Pol et al. 2006), theory predicts that variation in developmental environments could alternatively induce compensation or trade‐offs between fitness components (Metcalfe and Monaghan 2001; Moore and Martin 2019). Whether carryover effects on reproduction compound or buffer against the potentially lethal effects of global warming may crucially shape future extinction risk (Norris 2005; O'Connor and Cooke 2015).

BOX 1 **A Hypothesis Framework for Carryover Effects.**

Carryover effects occur when the environmental conditions experienced early in life (e.g. as a developing embryo or juvenile prior to adulthood) affect an individual's performance in subsequent life stages (Moore and Martin 2019; O'Connor et al. 2014). To characterise the types of carryover effects shaping performance traits, factorial experiments can test for specific patterns of performance across different combinations of developmental and adult environmental treatments. These carryover effects are mediated by developmental plasticity in morphological, physiological, and/or behavioural traits. Consequently, carryover effects could generate selection on these plastic traits to further exploit or ameliorate the fitness impacts of developmental plasticity (Moore and Martin 2019). Below, we outline the predictions supporting different forms of carryover effects on adult performance and the consequences of carryover effects for selection on adult traits.

*Silver spoon effects*: Silver spoon effects occur when developing under specific conditions enhances or hinders performance across all environments in later life stages (Lindström 1999; Van De Pol et al. 2006). Silver spoon effects are supported if one developmental treatment increases average performance across all adult treatments. Silver spoon effects could also be sex‐specific, with effects of developmental treatments that differ in magnitude and/or sign between males and females.

*Predictive adaptive plasticity*: Predictive adaptive plasticity allows individuals to achieve higher adult performance in conditions that reflect their early life experiences, and therefore typically evolves when variation in developmental environments predicts variation in adult environments (Bateson et al. 2014). Predictive adaptive plasticity is supported by significant interactions between developmental and adult treatments. For example, individuals that experienced relatively hot developmental treatments should achieve higher performance at relatively hot adult treatments. Predictive adaptive plasticity can also be sex‐specific; for instance, if only one sex's developmental treatment predicts its optimal adult treatment. Because reproduction requires performance by both sexes, adaptive plasticity (or a lack of plasticity) in one sex could even bias the sensitivity of reproduction to adult treatments in both sexes (Leith et al. 2021).

*Assortative reproduction*: Reproductive performance may be hindered if mating partners developed under different environmental conditions, as these individuals may express incompatible reproductive phenotypes (West‐Eberhard 2005). Carryover effects leading to assortative reproduction by developmental environment are supported by significant interactions between female and male developmental treatments, with reduced performance when males and females are from different treatments. Assortative reproduction carryover effects can be asymmetric; for example, if performance decreases when males from cold developmental treatments are paired with females from warm developmental treatments, but not vice versa.

*Consequences for selection on adult traits*: If carryover effects shape selection on adult phenotypes, then an individual's developmental environment and/or the developmental environment of their mates should influence (i) the expression of adult traits, and (ii) if and how developmentally plastic traits affect adult performance. For instance, optimal developmental environments may enable more individuals to express high‐performance adult phenotypes, leading selection to act in the same direction as plasticity in this environment (Moczek et al. 2011; West‐Eberhard 2005). When a developmental environment causes individuals to express maladaptive phenotypes, selection should instead act in the opposite direction of developmental plasticity. For instance, potential mates that developed in different environments may express more divergent reproductive phenotypes that are less compatible compared to potential mates that developed in the same environment (West‐Eberhard 2005). As a result, selection should be stronger when mates develop in mismatched environments, favouring individuals with traits that resemble the phenotypes expressed in their mate's developmental environment.

Carryover effects from embryonic and juvenile temperatures are widespread and can shape lifelong survival, adult behaviour, and reproductive performance in many ways (Kefford et al. 2022; Ma et al. 2021). Developmental temperatures can affect both average reproductive performance and the sensitivity of reproduction to adult temperatures (Abram et al. 2017). For example, optimal developmental temperatures can increase overall adult fecundity (silver spoon effect, Box 1; Min et al. 2021; Scharf et al. 2015; Vasudeva 2023), whereas suboptimal developmental temperatures can exacerbate reduced fecundity at suboptimal adult temperatures (Velikaneye and Kozak 2024). In other cases, individuals that survive stressful developmental temperatures can recover upon adulthood (Sales et al. 2021), or the adult temperatures where reproductive performance peaks may shift to match prevailing developmental temperatures (predictive adaptive plasticity, Box 1; Macchiano et al. 2023; Rebolledo et al. 2021; Slotsbo et al. 2016; Vasudeva et al. 2019). Different types of carryover effects may even shape different stages of reproduction such that developmental temperatures affect gametogenesis, mating behaviour, fertilisation, and parental care in distinct ways (Dougherty et al. 2024; Leith et al. 2021; Pilakouta and Baillet 2022; Sidhu et al. 2024).

Sex‐specific plasticity can have important reproductive consequences, since mating and fertilisation require coordination between males and females. Sexes typically differ in the thermal sensitivity of adult performance (Chatten et al. 2025; Pottier et al. 2021) and heightened thermal sensitivity in one sex can disproportionately shape the thermal sensitivity of mating rates between sexes (Brandt et al. 2018; Leith et al. 2020, 2021; Macchiano et al. 2023; Pembury Smith et al. 2025). Moreover, sex‐specific effects of developmental temperatures on sexual signals and mating preferences (Beckers and Schul 2008; Lis et al. 2020; Macchiano et al. 2023; Singh et al. 2020) could disrupt mate localization, species recognition, and the ability to choose beneficial mates in altered thermal environments (Tuomainen and Candolin 2011). Even just before and during copulation, sex‐specific plasticity in body size (Lafuente et al. 2018; Singh et al. 2020) and genitalia (Berger et al. 2011; Blanckenhorn and Hellriegel 2002; Fischer et al. 2003; Iglesias‐Carrasco et al. 2020; Klepsatel et al. 2019; McDonald et al. 2018; Vasudeva et al. 2014, 2019) can hinder genitalia coupling and sperm transfer (Eberhard et al. 1998; Holwell et al. 2010; Simmons 2014). By contrast, congruent plasticity between sexes can maintain reproduction when temperatures vary between generations (Vasudeva et al. 2019) or facilitate assortative mating by developmental environment (Box 1; Beckers and Schul 2008; West‐Eberhard 2005). Recent conceptual and computational advancements predict that these reproductive consequences of sex‐specific plasticity should scale up to shape population growth rates and the outcomes of natural selection, but tests of these hypotheses are rare (Box 1; Forsman 2018; Hangartner et al. 2022; Moore and Martin 2019; Stillwell et al. 2010).

Here, we tested how carryover effects from juvenile temperatures influence multiple stages of reproduction and examined the consequences of carryover effects for selection on adult phenotypes and population growth rates. Our study focused on *Enchenopa binotata* treehoppers, a phytophagous insect that experiences highly variable temperatures during development and adulthood (Jocson et al. 2019; Leith et al. 2024). We reared insects at two developmental temperatures (21°C or 26°C) and staged mating interactions between adults from the same or different developmental treatments. The mating interactions occurred across a continuous range of temperatures (28°C–33°C), allowing us to test how developmental temperatures affected overall performance and the sensitivity of reproduction to thermal variation during adulthood. We quantified changes in mating likelihood, fertility (sperm transferred and egg laying likelihood), and fecundity (number of eggs laid) and hypothesized that changes in performance at each stage of reproduction would be associated with mismatches in male–female morphology driven by sex‐specific plasticity. Finally, we developed a population dynamic model and parameterized it using our experimental results. This model evaluated the alternative hypotheses that carryover effects on each stage of reproduction culminate to either intensify or buffer against reduced survival in warming environments.

## 2 Materials and Methods

### 2.1 Study System

We studied *Enchenopa binotata* treehoppers that have specialised in the woody shrub, *Ptelea trifoliata* (Sullivan‐Beckers and Cocroft 2010; Wood 1980). *Enchenopa binotata* spends their entire life cycle on their host plant species, including overwintering as eggs in the stems, developing as juveniles during late spring, and mating as adults from mid to late summer (Sullivan‐Beckers and Cocroft 2010). We can therefore reliably link the effects of host plant temperatures on treehopper performance in the laboratory to natural variation in microclimates and lifelong treehopper fitness (Jocson et al. 2019; Leith et al. 2024; Macchiano et al. 2023).

### 2.2 Rearing Design

We collected wild *E. binotata* as 1st and 2nd instar juveniles in Columbia, Missouri, USA (38.924925, −92.318294) during the first week of May 2021 and May 2022. We sampled multiple trees and branches to account for genetic variation (Rebar and Rodríguez 2014). We reared the juveniles in a laboratory in St. Louis, MO, USA, where they were randomly assigned to a developmental temperature of 21°C or 26°C (*T*_dev_). These *T*_dev_ fall within the natural range of microclimates where juveniles develop until adulthood and reflect year‐to‐year differences in developmental microclimates at the field site (see Supporting Information; Figures S1 and S2; Macchiano et al. 2023). We placed approximately 125 juveniles on each of 16 potted and netted host plants in 2021 (eight plants for each *T*_dev_ treatment) and on each of 14 potted and netted host plants in 2022 (seven plants for each *T*_dev_ treatment). Rearing plants were placed in custom wooden incubators with glass doors (Macchiano et al. 2023) set to either 21°C or 26°C and 45% relative humidity with a 100 W full spectrum LED plant grow light (14:10 h light: dark photoperiod) mounted outside of each incubator. At adulthood, we moved the plants to four Darwin Chambers incubators (PG034) set to 23.5°C (the midpoint between the *T*_dev_ treatments) and 45% relative humidity. Additional details on the rearing protocol are provided in the Supporting Information.

### 2.3 Characterising Carryover Effects on Multiple Stages of Reproduction

We tested for carryover effects on mating likelihood, fertility, and fecundity by staging mating trials at sexual maturity (6 weeks into adulthood at the onset of female mating receptivity; Rodríguez and Cocroft 2006). In 2021, we paired males and females from either the same or different *T*_dev_ treatments and tested how developmental and adult mating temperatures affected mating likelihood and sperm transfer. In 2022, we only paired males and females from the same *T*_dev_ and tested how developmental and mating temperatures affected mating likelihood, egg‐laying likelihood, and total eggs laid. Our results from the 2021 experiment indicated low mating and fertilisation success for pairs from different *T*_dev_ treatments (see Results), which would have limited our sample size for fecundity. Moreover, pairs from similar *T*_dev_ are likely most common in wild *E. binotata* , given that developmental microclimates in the field show limited variation across individual plants within a year and significant variation across years (see Supporting Information). Only using matched *T*_dev_ treatments between sexes in the 2022 trials therefore prioritised the most ecologically realistic relationships between survival and reproduction under warming. This approach was key for our population dynamic models (see below), which were predominantly informed by the 2022 mating trials. Before each mating trial, we randomly paired a male and female from different rearing plant replicates and assigned the pair to one of the three target testing temperatures that we used as set points for the thermostats in the testing incubators (i.e., mating temperatures, *T*_mate_: 23°C, 28°C or 33°C). We recorded *T*_mate_ as the actual temperature at the time of mating, or at the end of the trial if mating did not occur, at a resolution of 0.1°C. The *T*_mate_ values thus varied continuously from 22.8°C to 35°C, reflecting natural variation in microclimate temperatures on host plants during peak mating season (Leith et al. 2024). Testing across this range is sufficient to detect variation in the optimal temperature for mating likelihood (Fowler‐Finn et al. 2025; Leith et al. 2020; Macchiano et al. 2023). Moisture released from the potted testing plants kept the testing environments at approximately 45% relative humidity regardless of *T*_mate_ treatment.

The mating trials followed established protocols for assaying thermal sensitivity in *E. binotata* mating behaviour (Leith et al. 2020; Macchiano et al. 2019, 2023; Sasson et al. 2022). We first acclimated individuals for 20 min in incubators at the assigned *T*_mate_. Then, we placed the female onto the main stem of the testing plant 15 cm above the soil. After the female acclimated for 5 min, we introduced the male approximately 10 cm below the female. We recorded whether mating occurred to estimate mating likelihood and allowed the pair to freely interact until they finished mating, or after 4 h if mating did not occur (Leith et al. 2020; Macchiano et al. 2023). We recorded mating latency and duration when mating occurred, since mating duration can affect fecundity in *E. binotata* (Cirino et al. 2024), and ended the trial once the pair naturally separated. All individuals were used only once and males were euthanized following trial completion. Overall, 253 mating trials were included in our analysis in 2021 and 169 mating trials were included in our analysis in 2022 (see Table S1 for sample size in each *T*_dev_–*T*_mate_ treatment combination).

To quantify sperm transfer in 2021, we euthanized and preserved mated females by freezing them at −20°C immediately following trials, then dissected sperm from their bursa copulatrix (organ where sperm is stored immediately after copulation before transfer to the spermatheca). To quantify egg laying likelihood and total eggs laid in 2022, we placed mated females individually on new netted plants and maintained them outside for overwintering. After all females senesced, we determined whether females laid at least one egg and counted the number of eggs in laid egg masses (following Fowler‐Finn et al. 2018). Egg masses are easily identified by their white waxy coating. See Supporting Information for further details on quantifying fertility and fecundity.

We characterised carryover effects at each stage of reproduction (mating likelihood, mating latency, mating duration, sperm transferred, egg laying likelihood, and total eggs laid; Box 1) using a series of generalised linear models (*stats*, R v4.2.0; R Core Team 2022) and visualised all results with *ggplot2* (Wickham 2016). See Supporting Information for details on the effect structures, error distributions, and link functions for each model.

### 2.4 Linking Carryover Effects to Plasticity in Reproductive Morphology

To test if morphological plasticity underlies carryover effects on reproduction (Box 1), we first measured a series of adult morphological traits for the individuals in the 2021 mating trials. We measured three traits representing body size (pronotum length, face length, and the length of right femur II; Rodríguez and Al‐Wathiqui 2012, 2011), two traits representing female genitalia (ovipositor traits 1 and 2), and five traits representing male genitalia (aedeagus traits 1–3 and style traits 1 and 2). See Supporting Information for details on morphometrics.

To test for plasticity in each morphological trait in response to developmental temperature (*T*_dev_), we fit linear mixed effects models (*lme4*; Bates et al. 2015) with each trait as a response variable, *T*_dev_ as a fixed effect, and rearing plant replicate as a random effect. We fit separate models for male and female traits and assessed significance with *F* tests. Supplementary analyses showed strong hypoallometry and no developmental plasticity in the scaling relationships between genitalia and body size, suggesting selection for precise coupling of the focal genital traits (see Supporting Information, Table S6; Eberhard et al. 1998). Next, we tested if *T*_dev_ affected relationships between morphology and reproductive performance (i.e., selection on morphology; Box 1) using separate generalised linear models for each of the four *T*_dev_ treatment combinations. Mating likelihood and sperm transfer were fit as response variables, with effects of *T*_mate_ and the traits that showed plasticity in response to *T*_dev_ (female face length, ovipositor trait 1, and aedeagus trait 3; see Supporting Information and Tables S8 and S9 for model details). We determined that selection on a morphological trait was stronger in a *T*_dev_ treatment if the standardised parameter estimate of the trait effect exceeded the 95% confidence intervals of the effects in other *T*_dev_ treatments.

### 2.5 Modelling the Impacts of Carryover Effects on Population Growth Rate

#### 2.5.1 Mathematical Model

We developed a mathematical model in R (R Core Team 2022) that incorporates the direct and carryover effects of temperature during development (*T*_dev_) and at the time of mating (*T*_mate_) and predicts *E. binotata* population growth rate over the course of one generation. Because female mating in *E. binotata* is not limited by the availability of males (Sullivan‐Beckers and Cocroft 2010), our model considered only female fitness and focused on the processes shaping female fecundity in our 2022 experiment. We modelled population growth rate using the five parameters that showed significant effects on reproductive performance (see Results). Females mate successfully with a probability of *P*_mate_. After mating, females lay eggs with probability *P*_egg_. If females lay eggs, the average number of eggs laid over their lifetime is *E*. The sex ratio of the hatching juveniles is *S*, and the probability of surviving from the egg stage until moult to adulthood is *μ*. We split *μ* into three components: egg hatching rate (*μ*_egg_), juvenile survival rate (*μ*_juv_), and adult survival to sexual maturity (*μ*_adult_).

We used the following model to simulate the effects of temperature during development and mating on *E. binotata* population growth rate. With *N*_*t*_ as the number of females at time *t*, the change in population size can be modelled as:

$$N_{t+1}=N_{t}\cdot P_{\text{mate}}\left(T_{\text{mate}}\right)\cdot P_{\mathrm{egg}}\left(T_{\mathrm{dev}}\right)\cdot E\cdot S\cdot \mu_{\mathrm{egg}}\cdot \mu_{\mathrm{juv}}\left(T_{\mathrm{dev}}\right)\cdot \mu_{\text{adult}}$$

where $P_{\text{mate}}\left(T_{\text{mate}}\right)$ indicates that $P_{\text{mate}}$ depends on $T_{\text{mate}}$, and $P_{\mathrm{egg}}\left(T_{\mathrm{dev}}\right)$ and $\mu_{\mathrm{juv}}\left(T_{\mathrm{dev}}\right)$ indicates that $P_{\mathrm{egg}}$ and $\mu_{\mathrm{juv}}$ depend on $T_{\mathrm{dev}}$. The population growth rate λ is:

$$\lambda =\frac{N_{t+1}}{N_{t}}$$

where λ = 1 indicates population stasis, λ > 1 indicates population growth, and λ < 1 indicates population decline. We simulated λ for populations of 100 females across for each combination of *T*_dev_ (21°C or 26°C) and *T*_mate_ (every 1°C between 10°C and 40°C), encompassing the full range of potential mating temperatures encountered in the field (Jocson et al. 2019; Leith et al. 2024). The model determined whether each female mated and oviposited at least one egg by randomly drawing values from a uniform distribution between 0 and 1; a female mated or laid at least one egg if the random number was less than *P*_mate_ or *P*_egg_, respectively. The number of offspring produced by each female that mated and then oviposited was drawn from a Poisson distribution (mean = expected lifetime egg production (*E*)).

#### 2.5.2 Estimating Model Parameters

We used generalised linear models and the data from the 2022 mating trials to parameterize effects of *T*_dev_ and *T*_mate_ on each stage of reproduction. We used a binomial error distribution for estimating *P*_mate_ and *P*_egg_, and a gaussian error distribution for estimating log(*E*). We included a quadratic term to estimate the effect of *T*_mate_ on *P*_mate_ since *P*_mate_ is highest at intermediate values of *T*_mate_:

$$P_{\text{mate}}=\frac{1}{1+\exp\left(21.66-1.61\cdot T_{\text{mate}}+0.03\cdot {T_{\text{mate}}}^{2}\right)}$$

The *T*_mate_ estimate assumes that females that have not mated after 4 h will not mate even if they encounter another male at the same temperature, since our mating trials were 4 h long and the mean latency to mate when mating occurs is around 1.5 h (see Results; Leith et al. 2020; Macchiano et al. 2023). Parameterizing the quadratic effect of *T*_mate_ on *P*_mate_ using the data from the 2021 mating trials produced similar effects on the relationships between population growth rates across *T*_dev_ (Figure S4). We used the model estimates from an effect of *T*_dev_ on *P*_egg_, where *P*_egg_ = 0.85 when *T*_dev_ = 21°C and *P*_egg_ = 1.0 when *T*_dev_ = 26°C. Because we did not detect significant effects of *T*_dev_ or *T*_mate_ on *E*, we used the median value of *E* = 71 eggs. We did not directly collect data on the sex ratio *S* of the hatching juveniles nor on the probability of surviving from eggs to the adult stage *μ*. However, a previous field study (Leith et al. 2024) suggests that adult sex ratios are approximately equal at the start of the mating season (*S* = 0.5). To estimate *μ*, we estimated *μ*_egg_ = 0.2, *μ*_juv_ = 0.4, and *μ*_adult_ = 0.9 based on previous work in *E. binotata* and other ecologically similar treehopper species (Caceres‐Sanchez et al. 2017; Morales et al. 2022; Wood and Guttman 1982; Zink 2003), resulting in an overall *μ* = *μ*_egg_ · *μ*_juv_ · *μ*_adult_ = 0.07. Although juvenile mortality was not directly measured in 2022, we used the ratio of adults that emerged in each *T*_dev_ treatment in 2021 and in a previously published study (Macchiano et al. 2023) to estimate the impacts of *T*_dev_ on survival. We found 12% fewer adults surviving at *T*_dev_ = 26°C (676 total adults) compared to *T*_dev_ = 21°C (768 total adults) in our 2021 experiment, despite starting with approximately equal numbers. We explored the effects of different levels of reduced juvenile survival at *T*_dev_ = 26°C to account for uncertainty in this effect (see below).

#### 2.5.3 Modelling Scenarios

We examined three scenarios where *T*_dev_ affected juvenile survival (*μ*_juv_): no effect of *T*_dev_ on juvenile survival (*μ*_juv_ = 0.4), a moderate effect (*μ*_juv_ = 0.43 when *T*_dev_ = 21°C and 0.37 when *T*_dev_ = 26°C), or a severe effect (*μ*_juv_ = 0.48 when *T*_dev_ = 21°C and 0.32 when *T*_dev_ = 26°C). The moderate survival effect was calculated based on typical juvenile survival in nature (*μ*_juv_ = 0.4; see above) and the ratio of surviving adults in the 21°C versus 26°C *T*_dev_ treatments in the 2021 mating trials (768:676 adults). The severe effect was calculated with the same method, but using the ratio of surviving adults in a previous study with the same *T*_dev_ treatments (117:79 adults; Macchiano et al. 2023). Finally, we examined whether the carryover effect of *T*_dev_ on *P*_egg_ counteracted the effects of *T*_dev_ on *μ*_juv_ by defining *P*_egg_ = 0.93 at both values of *T*_dev_ in each survival scenario and comparing the outcomes of these population models to models that included an effect of *T*_dev_ on *P*_egg_. We repeated the simulation 500 times for all six population models (three survival scenarios, either with or without carryover effects of *T*_dev_ on *P*_egg_) and calculated the means and 95% confidence intervals for the distributions of predicted λ values.

## 3 Results

### 3.1 Characterising Carryover Effects on Multiple Stages of Reproduction

#### 3.1.1 Effects of *T*_dev_ and *T*_mate_ on Mating Likelihood

**FIGURE 1:**
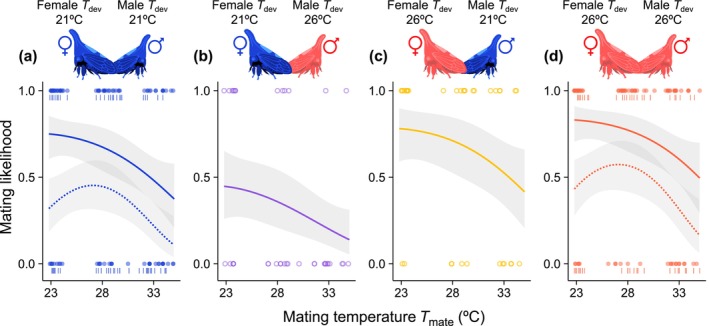
Effects of *E. binotata* developmental temperatures (*T*_dev_) and adult mating temperatures (*T*_mate_) on mating likelihood in the 2021 mating trials (points and solid lines) and 2022 mating trials (bars and dashed lines, a and b only). Point colours indicate female and male *T*_dev_, where blue is 21°C and 21°C (a), open purple is 21°C and 26°C (b), open yellow is 26°C and 21°C (c), and red is 26°C and 26°C (d), respectfully. Fitted curved and 95% CI bands were determined from GLMs. Mating likelihood decreased when females reared at 21°C were paired with males reared at 26°C. Mating likelihood was also highest at intermediate *T*_mate_, reflecting previous results across a broader *T*_mate_ range (Figure S3; Macchiano et al. 2023), and the optimal *T*_mate_ appeared slightly warmer in 2022 than in 2021 (a, d).

An interaction between female and male *T*_dev_ in 2021 suggests asymmetric assortative mating ($\chi_{1}^{2}$ = 7.09, *p* = 0.0078; Table S3; Box 1), with reduced mating when females reared at 21°C were paired with males reared at 26°C (Figure 1). Mating likelihood peaked between 24°C and 28°C *T*_mate_, indicated by a negative linear effect of *T*_mate_ in 2021 ($\chi_{1}^{2}$ = 13.55, *p* = 0.0002) and a quadratic *T*_mate_ effect in 2022 ($\chi_{1}^{2}$ = 5.59, *p* = 0.0180; Figure 1). Notably, this quadratic *T*_mate_ effect closely resembled a published mating likelihood thermal performance curve using the same *T*_dev_ treatments but a broader range of *T*_mate_ (Figure S3; Macchiano et al. 2023). No interaction between *T*_dev_ and *T*_mate_ suggests no predictive adaptive plasticity in mating likelihood (Box 1; Table 1). Finally, mating duration decreased at hotter *T*_mate_, but *T*_dev_ did not affect mating duration (Table S4) and neither *T*_dev_ nor *T*_mate_ affected mating latency (Table S4).

#### 3.1.2 Effects of *T*_dev_ and *T*_mate_ on Sperm Transfer

**FIGURE 2:**
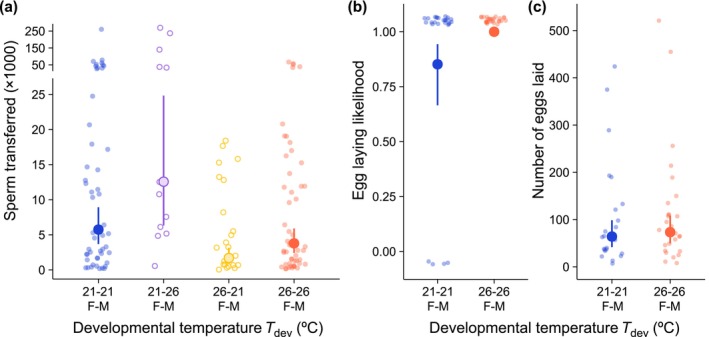
Effects of *E. binotata* developmental temperatures (*T*_dev_) on (a) sperm transferred during mating in the 2021 trials, and (b) egg laying likelihood and (c) total eggs laid in the 2022 mating trials. Large points show estimated marginal means from GLMs ±95% CI. Point colours indicate female and male *T*_dev_, where blue is 21°C and 21°C, open purple is 21°C and 26°C, open yellow is 26°C and 21°C, and red is 26°C and 26°C, respectfully. (a) Sperm transfer increased when females developed at 21°C and when males developed at 26°C, regardless of pairing. (b) Egg laying likelihood increased when treehoppers developed at 26°C. (c) Total eggs laid was not affected by *T*_dev_. Adult mating temperature (*T*_mate_) did not affect sperm transfer, egg laying likelihood, or total eggs laid.

Females received more sperm if they developed at 21°C *T*_dev_ ($\chi_{1}^{2}$ = 12.71, *p* = 0.0004), but males transferred more sperm if they developed at 26°C *T*_dev_ ($\chi_{1}^{2}$ = 5.37, *p* = 0.0205; Table S3; Figure 2a), indicating sex‐specific silver spoon effects on sperm transfer (Box 1). Sperm transfer was not affected by *T*_mate_ (nor a *T*_dev_ by *T*_mate_ interaction; Table S3) and therefore did not show predictive adaptive plasticity (Box 1).

#### 3.1.3 Effects of *T*_dev_ and *T*_mate_ on Egg Laying Likelihood and Fecundity

Four out of 27 mated females in the 21°C *T*_dev_ treatment did not produce eggs (Figure 2b), but mated females always produced eggs when *T*_dev_ was 26°C, indicating a silver‐spoon effect on female fertility ($\chi_{1}^{2}$ = 6.16, *p* = 0.0131; Table S3; Figure 2b; Box 1). However, *T*_dev_ did not affect the number of eggs laid (Table S3; Figure 2c). Egg‐laying likelihood and the number of eggs laid were not affected by *T*_mate_ (nor a *T*_dev_ by *T*_mate_ interaction; Table S3) and therefore did not show predictive adaptive plasticity (Box 1).

### 3.2 Linking Carryover Effects to Plasticity in Reproductive Morphology

**FIGURE 3:**
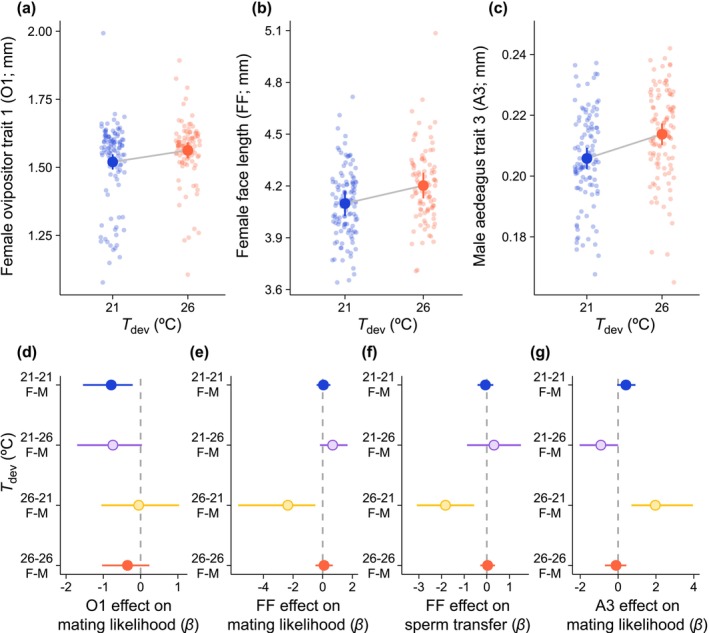
Effects of *E. binotata* developmental temperatures (*T*_dev_) on (a–c) female and male morphological traits related to body size and genitalia, and (d–g) the effects of morphological variation on mating success and sperm transfer in the 2021 mating trials. In (a–c), blue points indicate 21°C *T*_dev_ and red points indicate 26°C *T*_dev_. Large points in (a–c) show estimated‐marginal means from LMMs ±95% CI. Points in (d–g) show standardised parameter estimates (*β*) ± 95% CI from GLMs.

**FIGURE 4:**
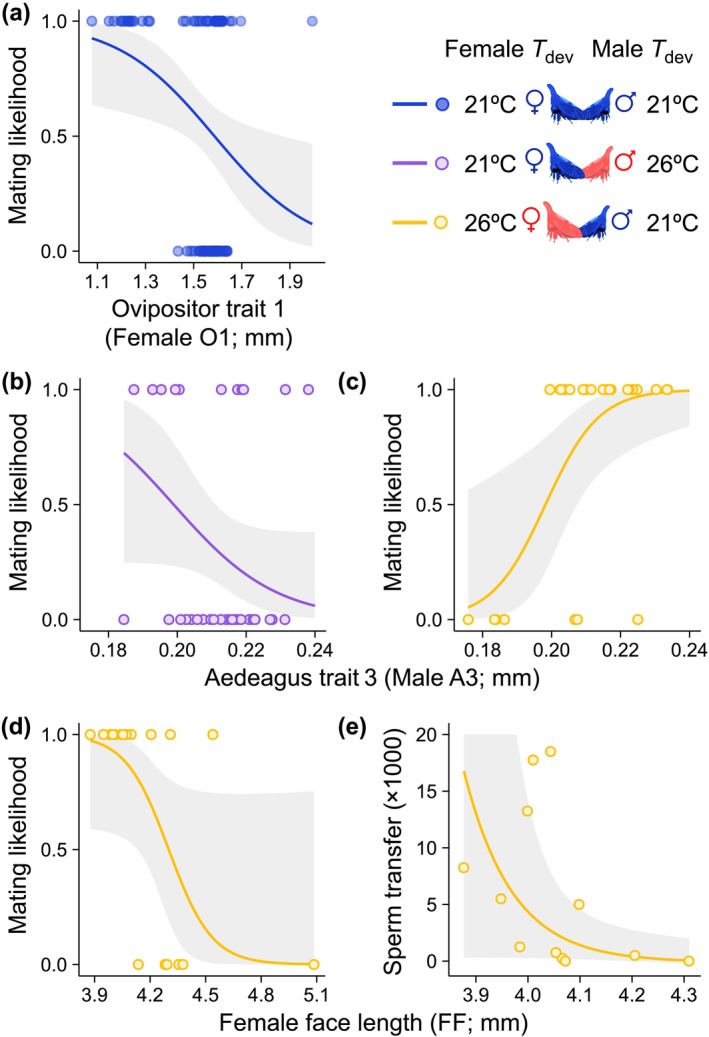
Changes in selection on *E. binotata* morphology across developmental temperatures (*T*_dev_). Colours indicate female and male *T*_dev_ (blue is 21°C and 21°C, purple is 21°C and 26°C, and yellow is 26°C and 21°C). Lines and bands are estimated fits ±95% CI from GLMs. (a) Females with smaller ovipositors mated more when *T*_dev_ was 21°C for both sexes. (b) Males with smaller aedeagi mated more when *T*_dev_ was 21°C for females and 26°C for males. When *T*_dev_ was 26°C for females and *T*_dev_ 21°C for males, mating likelihood increased for the smallest females (c) and for males with larger aedeagi (d), and smaller females received more sperm (e).

Hotter *T*_dev_ led to larger female ovipositors (ovipositor trait 1; *F* = 5.17, *p* = 0.0395), larger female body sizes (female face length; *F* = 4.61, *p* = 0.0498), and larger male aedeagi (aedeagus trait 3; *F* = 11.75, *p* = 0.0043; Table S7; Figure 3). Developmental temperature also affected the relationships between these traits and reproductive performance. Ovipositor size only affected mating likelihood when *T*_dev_ was 21°C for both sexes, with smaller ovipositors conferring increased mating success ($\chi_{1}^{2}$ = 7.71, *p* = 0.0055; Figures 3d and 4a; Table S8).

When *T*_dev_ was 21°C for females and 26°C for males (the treatment with relatively small females and large males), males with smaller aedeagi were more likely to mate ($\chi_{1}^{2}$ = 3.86, *p* = 0.0494; Table S8; Figures 3g and 4b). When *T*_dev_ was 26°C for females and *T*_dev_ 21°C for males (the treatment with relatively large females and small males), males with larger aedeagi were more likely to mate ($\chi_{1}^{2}$ = 11.39, *p* = 0.0007; Table S8; Figures 3g and 4c) and the smallest females were more likely to mate ($\chi_{1}^{2}$ = 7.71, *p* = 0.0055; Table S8; Figures 3g and 4d). Smaller females also received more sperm in this *T*_dev_ treatment combination ($\chi_{1}^{2}$ = 8.03, *p* = 0.0046; Table S9; Figures 3f and 4e). Overall, predicted selection on male and female morphology was stronger in the mismatched *T*_dev_ treatment combinations (Figure 3d–g) that generated the greatest morphological mismatches between sexes (Figure 3a–c), and predicted selection in these treatments favoured less divergent phenotypes between sexes (Figure 4b–e).

### 3.3 Modelling the Impacts of Carryover Effects on Population Growth Rate

**FIGURE 5:**
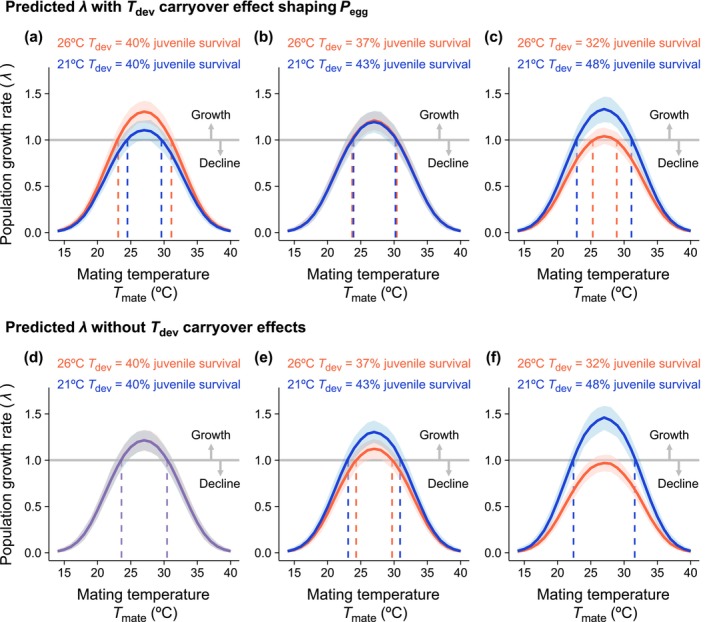
Simulation model estimating variation in *E. binotata* population growth rate (*λ*) across developmental temperatures (*T*_dev_) and adult mating temperatures (*T*_mate_). In all cases, mating likelihood depends on *T*_mate_. In (a–c), the probability for mated females to lay eggs depended on *T*_dev_. In (d–f), the probability for mated females to lay eggs was fixed at 0.93. The negative effect of hotter *T*_dev_ on juvenile survival strengthens from left to right, varying between no effect (a, d), a moderate effect (b, e), or a severe effect (c, f). The dashed vertical lines bound the *T*_mate_ range leading to positive population growth. Curves and bands show the means and 95% CI for *λ* across 500 simulations. Blue curves indicate 21°C *T*_dev_ and red curves indicate 26°C *T*_dev_.

Our models predict population declines (λ < 1) when mating interactions occur at thermal extremes (Figure 5). Carryover effects of *T*_dev_ on egg laying likelihood (*P*_egg_) and direct effects of *T*_dev_ on juvenile survival to adulthood (*μ*_juv_) culminated to shape the peak population growth rate and the range of adult *T*_mate_ where population size increases (λ > 1). Specifically, when *T*_dev_ has no effect on juvenile survival, warmer *T*_dev_ causes higher peak growth rates and broadens the range of *T*_mate_ across which population size increases due to the silver spoon effects of warmer *T*_dev_ on egg laying likelihood (Figure 5a). However, our models show minimal differences in population growth rate across *T*_mate_ when *T*_dev_ only moderately reduces juvenile survival (Figure 5b). By contrast, peak growth rates increase at warmer *T*_dev_ and population growth is positive across a broader range of *T*_mate_ when *T*_dev_ more severely reduces juvenile survival (Figure 5c). Positive carryover effects on reproduction at warmer *T*_dev_ could therefore stabilise or even reverse the deleterious effects of warmer *T*_dev_ on survival. Supporting this, removal of the carryover effect of *T*_dev_ on egg‐laying likelihood (*P*_egg_) has two main consequences: hotter *T*_dev_ always reduces population growth rate (Figure 5e,f) and population declines occur across all values of *T*_mate_ when hotter *T*_dev_ severely reduces *μ*_juv_ (Figure 5f).

## 4 Discussion

Vulnerability to global warming depends on the thermal sensitivity of both survival and reproduction (Johnson et al. 2023; Kingsolver and Buckley 2020). Despite widespread carryover effects on performance in both contexts, current predictive models typically ignore changes in multiple fitness components and their cumulative impacts on population persistence (Sgrò et al. 2016; Sinclair et al. 2016). We show that even relatively weak carryover effects increasing fertility can help buffer *E. binotata* against reduced survival at elevated developmental temperatures, potentially averting population declines in the wake of global warming. To be sure, more severe temperature increases than those examined here would likely reduce fertility, and relationships between the thermal limits of survival and reproduction vary considerably among taxa (Parratt et al. 2021; Walsh et al. 2019). Our key finding is that thermal variation across life stages can generate compensation among different fitness components, suggesting multiple pathways toward persistence in warming climates. Population dynamics estimated from lethal stress alone, or without considering carryover effects across life stages, may be unreliable for predicting extinction risk.

Conflicts in the thermal sensitivity of male and female performance may threaten persistence in changing climates (García‐Roa et al. 2020; Leith et al. 2021, 2022). In some cases, temperature change can directly increase the harm inflicted on one sex during mating (Breedveld et al. 2023; García‐Roa et al. 2019), potentially exacerbating extinction risk (Flintham et al. 2023). Our results show how differences in male and female developmental temperatures could also create apparent conflict between the sexes and fitness components beyond affecting direct harm during mating. Mating success was lowest when females reared at cooler temperatures were paired with males reared at hotter temperatures, but the males of these pairs also transferred the most sperm during mating; sex‐specific carryover effects that enhance mating likelihood in females may therefore trade off with sperm transfer performance or limit mating to males that also produce more sperm.

Our population dynamic models revealed that adult thermal niche breadth (i.e., the range of adult mating temperatures that confers positive population growth, *sensu* Hutchinson 1957) depends on the balance between survival and carryover effects on fertility. When survival did not differ across environments, hotter developmental temperatures expanded the adult thermal niche (Figure 5a). Models exploring whether plasticity can buffer populations from global warming typically assess whether predictive adaptive plasticity in optimal temperature ranges or the skew of adult thermal performance curves tracks temperature shifts (Sinclair et al. 2016; Walsh et al. 2019). We instead demonstrate how carryover effects on only average fertility across all adult temperatures could still expand adult thermal niches and promote persistence in warming climates, since this enhanced fertility provides a population‐level fitness buffer at adult temperature extremities where mating activity is low. However, severely reduced survival in hotter developmental environments still offset increased fertility, narrowing the adult thermal niche (Figure 5c) and potentially limiting population viability. Although insects experience a vast range of microclimates at fine spatial scales (Caillon et al. 2014; Leith et al. 2024; Pincebourde and Woods 2020; Woods et al. 2015), the widest adult thermal niche identified by our model is already mostly unavailable to treehoppers in nature (approximately 23°C to 32°C; Figure 5; Leith et al. 2024). Moreover, treehoppers (and likely many other plant‐living insects) do not actively thermoregulate to maintain the optimal body temperatures for mating (Leith et al. 2024). Shifting or widening adult thermal niches to encompass hotter temperatures may therefore be necessary for populations to persist in changing climates (Kearney et al. 2009; Sunday et al. 2014).

Plasticity in key morphological traits likely contributed to assortative mating by developmental temperature and changes in selection on male and female reproductive morphology across thermal environments. Selection often favours the developmental canalization of genitalia and other morphological traits involved in copulation (Eberhard et al. 1998). Our results show that canalization could be further favoured by selection when differences in male and female developmental temperatures exacerbate morphological mismatches between sexes, allowing only males and females with the least divergent morphologies to reproduce (Box 1; Figure 3, Figure 4c–e). However, the selection landscape on thermal plasticity in morphology is likely more complex. Developing under cooler temperatures led females to produce smaller ovipositors that enhanced mating success (Box 1; Figure 3, Figure 4a), thereby exposing female genitalia to directional selection (Moczek et al. 2011; West‐Eberhard 2005). If small ovipositors compensate for detrimental changes in other copulatory traits under cool conditions (Morris and Rogers 2013), then selection may even favour the evolution of more pronounced plasticity in ovipositors to enable females to produce different adaptive morphological traits (or trait combinations) across developmental environments (Moore and Martin 2019).

Developmental plasticity in lethal thermal limits is insufficient for most organisms to fully accommodate global warming (Gunderson et al. 2017; Gunderson and Stillman 2015; Pottier et al. 2022). However, even without considering plasticity in lethal thermal limits, our findings show that any fertility benefits of elevated developmental temperatures could help offset typically higher mortality rates in warming climates. We also demonstrate how patterns of morphological plasticity underlying these carryover effects can shape selection on reproductive morphology. Whether reproductive traits ultimately evolve in response to such altered selection regimes will crucially affect whether organisms can adapt to global warming (Leith et al. 2022; Moore et al. 2021). Overall, accurately predicting vulnerability to global warming requires considering not only acute thermal sensitivity in multiple fitness components, but also long‐term plasticity in each fitness component due to temperature perturbations across the life cycle.

## Author Contributions

A.M., N.T.L., and K.D.F.‐F. designed the experiment. A.M., I.S., J.P.W., and N.T.L. collected the data. B.T. constructed the population model; B.T., N.L.T. and K.D.F.‐F. estimated model parameters from the data presented. N.T.L. performed the remaining statistical analysis and generated the figures. N.T.L., A.M., and K.D.F.‐F. wrote the manuscript, and B.T. provided editing suggestions. K.D.F.‐F. provided project management and funding.

## Conflicts of Interest

The authors declare no conflicts of interest.

## Supporting information


**Table S1:** Sample size (number of male–female pairs) for each developmental temperature (*T*_dev_) and adult mating temperature (*T*_mate_) treatment combination used in the 2021 and 2022 mating trials.
**Table S2:** Results from global generalised linear models testing the effects of female and male developmental temperature (*T*_dev_) and adult mating temperature (*T*_mate_) on each stage of reproductive success. Significant parameters are indicated in bold. Interactions and quadratic effects with *p* > 0.05 were removed from the final models.
**Table S3:** Results from generalised linear models testing the effects of female and male developmental temperature (*T*_dev_) and adult mating temperature (*T*_mate_) on each stage of reproductive success. The 95% confidence interval for the *T*_dev_ parameter estimate (*β*) could not be calculated for egg‐laying likelihood because all mated females laid eggs in the 26°C *T*_dev_ treatment (indicated by dashes). Significant parameters are indicated in bold. Interactions and quadratic effects with *p* > 0.05 were removed from the final models and are therefore not shown. We retained the non‐significant quadratic *T*_mate_ effect in the mating likelihood analysis so that the fitted *P*_mate_ curves across *T*_mate_ were comparable between years.
**Table S4:** Results from generalised linear models testing the effects of female and male developmental temperature (*T*_dev_) and adult mating temperature (*T*_mate_) on mating latency and duration. Significant parameters are indicated in bold. Interactions with *p* > 0.05 were removed from the final models and are therefore not shown.
**Table S5:** Results from post hoc contrasts of the estimated marginal means of mating likelihood across each male and female *T*_dev_ combination in the 2021 mating trials. Significant parameters are indicated in bold.
**Table S6:** Effects of rearing treatment on the allometric scaling of male and female morphological traits. We specified an offset slope of 1 for ln (Pronotum length) to test the null hypothesis that the scaling of each genitalia trait with pronotum length was isometric. Rearing plant was included as a random effect in all models. Results were obtained from linear mixed‐effects models using *F* tests and the Kenward‐Roger degrees of freedom approximation. Significant parameters are indicated in bold.
**Table S7:** Effects of developmental temperature (*T*_dev_) on male and female morphological traits. Rearing plant was included as a random effect in all models. The number of individuals measured in each treatment are listed under *n*. Results were obtained from linear mixed‐effects models using *F* tests and the Kenward‐Roger degrees of freedom approximation. Significant parameters are indicated in bold.
**Table S8:** Results of generalised linear models testing the effects of female and male morphological traits on mating likelihood across combinations of female and male developmental temperature (*T*_dev_). Mating temperature (*T*_mate_) was included as a covariate in all models. However, the parameter estimates for *T*_mate_ are not reported here for simplicity, as all trait effects on mating likelihood were analysed in separate models (see Methods for justification). Significant parameters are indicated in bold.
**Table S9:** Results of generalised linear models testing the effects of female and male morphological traits on sperm transfer across combinations of female and male developmental temperature (*T*_dev_). Significant parameters are indicated in bold.
**Figure S1:** Temperature regimes on plants where *E. binotata* were collected in the field. (a) Temperatures regimes measured during the developmental stage (May 1—May 30). (b) Temperature regimes measured during peak mating season (June 14—July 14). Curve colours represent temperatures measured on different collection plants. Solid curves represent temperatures measured in 2021, while dashed curves indicate temperatures measured in 2022. Curves were derived from a cyclic generalised additive mixed effects model.
**Figure S2:** Variation in temperature regimes within plants where *E. binotata* were collected in the field. Individual curves represent individual iButton temperature loggers. Curve colours represent temperatures measured on different collection plants. Solid curves represent temperatures measured in 2021, while dashed curves indicate temperatures measured in 2022. The top row show temperature regimes measured during the developmental stage (May 1—May 30). The bottom row shows temperature regimes measured during peak mating season (June 14—July 14). Curves were derived from cyclic generalised additive mixed effects models.
**Figure S3:** Mating likelihood in 2022 was highest at intermediate *T*_mate_. Reflecting previous results across a broader *T*_mate_ range. The black curve and points in show results from the 2022 mating trials in this study and the grey lines and points show results from an experiment performed in 2019 that tested mating likelihood across a wider range of mating temperatures (Macchiano et al. 2023). Lines and bands are estimated fits ±95% CI from GLMs.
**Figure S4:** Simulation model estimating variation in *E. binotata* population growth rate (*λ*) across developmental temperatures (*T*_dev_) and adult mating temperatures (*T*_mate_) where the effect of *T*_mate_ on mating likelihood was parameterized using data from the 2021 mating trials. In (a–c), the probability for mated females to lay eggs depended on *T*_dev_. In (d–f), the probability for mated females to lay eggs was fixed at 0.93. The effect of *T*_dev_ on offspring survival as juveniles varied between no effect (a, d), a moderate effect (b, e), or a severe effect (c and f). Curves and bands show the means and 95% CI for *λ* across 500 simulations. Blue curves indicate 21°C *T*_dev_ and red curves indicate 26°C *T*_dev_.

## Data Availability

The data and code are available at Figshare (Leith et al. 2025): https://figshare.com/s/d84135a57fe83dd8079b.
