# Supplementary material for: Carryover Effects on Reproduction Can Buffer Against Mortality‐Driven Population Declines at Elevated Developmental Temperatures
Source: Ecol Lett. 2025 Nov 11;28(11):e70264. doi: 10.1111/ele.70264 (PMC12605781; doi:10.1111/ele.70264)
Supplement: Supplementary file 1 — Table S1: Sample size (number of male–female pairs) for each developmental temperature (T dev) and adult mating temperature (T mate) treatment combination used in the 2021 and 2022 mating trials. Table S2: Results from global generalised linear models testing the effects of female and male developmental temperature (T dev) and adult mating temperature (T mate) on each stage of reproductive success. Significant parameters are indicated in bold. Interactions and quadratic effects with p > 0.05 were removed from the final models. Table S3: Results from generalised linear models testing the effects of female and male developmental temperature (T dev) and adult mating temperature (T mate) on each stage of reproductive success. The 95% confidence interval for the T dev parameter estimate (β) could not be calculated for egg‐laying likelihood because all mated females laid eggs in the 26°C T dev treatment (indicated by dashes). Significant parameters are indicated in bold. Interactions and quadratic effects with p > 0.05 were removed from the final models and are therefore not shown. We retained the non‐significant quadratic T mate effect in the mating likelihood analysis so that the fitted P mate curves across T mate were comparable between years. Table S4: Results from generalised linear models testing the effects of female and male developmental temperature (T dev) and adult mating temperature (T mate) on mating latency and duration. Significant parameters are indicated in bold. Interactions with p > 0.05 were removed from the final models and are therefore not shown. Table S5: Results from post hoc contrasts of the estimated marginal means of mating likelihood across each male and female T dev combination in the 2021 mating trials. Significant parameters are indicated in bold. Table S6: Effects of rearing treatment on the allometric scaling of male and female morphological traits. We specified an offset slope of 1 for ln (Pronotum length) to test the null hypothesis tha [file ELE-28-0-s001.docx]

**Title:** Carryover effects on reproduction can buffer against mortality-driven population declines at elevated developmental temperatures

**Authors:** Noah T. Leith, Anthony Macchiano, Brigitte Tenhumberg, Inaya Smith, Jake P. Woods, and Kasey D. Fowler-Finn

**Supporting information methods and results**

*Quantifying environmental temperatures during development and mating in the field.* We determined the range of temperatures *E. binotata* experience during development and the mating season at our collection site using Hygrochron DS1923-F5# iButton temperature loggers. We deployed loggers on four *Ptelea trifoliata* trees from which we collected the insects during both the developmental season (May 1 – May 30) and adult mating season (June 14 – July 14) in two subsequent years, 2021 and 2022. We deployed three loggers on each tree, set to record temperature every hour. All loggers were placed adjacent to egg masses or adjacent to egg mass scaring, indicating areas of the tree where egg masses were found in previous years. For each tree, one logger was placed at each of three positions, approximately 1, 1.5, and 2 meters above the ground.

We examined variation in temperature across collection plants, time (hour) of day, seasons (developmental versus mating), and years using a generalized additive mixed effects model with the R package *mgcv* (Wood 2022). The model included temperature as the response variable and a cyclic cubic spline term with 20 basis dimensions (k) for hour of day. We included collection plant (each plant including data from three loggers), season, and year as predictor variables. For each of these three predictor variables, we also included an interaction with that interacted with hour of day to produce eight separate curves that represent different combinations of plants, seasons, and years. We used k = 20 for the cyclic time curves because a model with k = 10 (default) did not produce sufficient smoothing (k-index < 1). Setting k = 20 was sufficient (k-index > 1) and increasing k beyond 20 prohibitively increased computational time. Comparing this global model with all interaction terms to models with all possible combinations of lower-order fixed effects and subsets of interaction terms showed that the global model was better supported than less complex models (ΔAICc = 51.04). We extracted eight curves of temperature across hour of day that represented data from each combination of collection plant, season, and year. We then visually compared the curves to assess how each factor generated variation in environmental temperatures.

Lastly, we assessed how temperatures varied across loggers within collection plants. We fit four separate models for each season and year combination (developmental/mating season and 2021/2022) due to convergence issues when fitting a single model with all covariates. All models included temperature as the response variable, logger as a factor predictor variable, a cyclic smoothed term with 20 basis dimensions for hour of day, and an interaction between logger and hour of day. We then visually compared curves for loggers within each combination of collection plant, season, and year.

Temperatures differed among plants in our collection site only within the hours of 08:00 to 14:00 (Fig. S1), with only small differences among plants at other times of day. Temperatures did not vary substantially among loggers within individual plants (Fig. S1). The average temperature difference between the developmental period (May 1-30) and the mating season (June 14 – July 14) was approximately 6.5ºC. From 2021 to 2022 there was an average increase in temperature of approximately 2.5ºC (Fig. S1). Warmer field temperatures during development before treehoppers were collected in 2022 may explain why the optimal adult mating temperature for mating likelihood was slightly warmer in 2022 compared to 2021 (Fig. 1a and 1d), reflecting predictive adaptive plasticity. However, we cannot decisively conclude that mating likelihood acclimated to early developmental temperatures because this difference in field temperatures across years is not replicated.

*Additional procedure information for rearing and mating trials.* We reared all treehoppers on *Ptelea trifoliata* that were potted simultaneously as saplings with no leaf buds in January of each testing year. We grew the saplings in a common greenhouse with the same watering schedule (2-3 times per week). Consequently, all rearing plants were the same age (four months since planting) and approximately the same size when juvenile treehoppers were placed on the plants. We controlled for plant quality by only using plants that produced leaves and did not show leaf rust or mold. Treehopper juveniles were collected from the field as 1^st^ or 2^nd^ instars, approximately one week after hatching. We reared juveniles to adulthood in the laboratory at their respective *T*_dev_ treatments. The duration of the developmental treatments varied from 2-3 weeks in the 26°C *T*_dev_ treatment to 3-4 weeks in the 21°C *T*_dev_ treatment, as individuals reared at 26°C molted to adulthood one to two weeks earlier than those reared at 21°C. We therefore staggered the mating trials so that age after maturity was consistent across *T*_dev_ treatments. In 2021, we started with mating trials between males and females both reared at 26°C *T*_dev_, followed by trials that paired males and females from different *T*_dev_ treatments, and then trials that paired males and females both reared at 21°C *T*_dev_. We did not pair males and females from different treatments in the 2022 mating trials, but we again started with pairs reared at 26°C and followed with pairs reared at 21°C. To prevent mating before testing, we transferred males and females to separate new plants upon the molt to adulthood. We identified individuals after adulthood by applying unique two-color combinations of non-toxic acrylic paint (Apple Barrel, multi surface, satin finish) on either side of the pronotum (Jocson *et al.* 2019; Leith *et al.* 2020; Macchiano *et al.* 2023; Sasson *et al.* 2022).

*Quantifying sperm transfer*. To test for carryover effects of developmental temperatures on sperm transfer, we dissected sperm from the bursa copulatrix (sperm storage organ) of mated females used in the 2021 mating trials. We placed the bursa copulatrix in a 1.5ml microcentrifuge tube with 75µl of 0.9% saline solution, crushed the sample with a pestle, and then added 25µl of 25% glutaraldehyde solution as a preservative. Next, we mixed 10µl of this sperm solution with 10µl of 0.5% Eosin staining dye and 2µl of 0.9% saline. We then pipetted 10µl of the resulting solution onto each side of an Improved Neubauer hemocytometer. The Neubauer hemocytometer has two 3mm-by-3mm square counting grids, both with nine 1mm-by-1mm subdivisions. For the sperm sample from each mated pair, we counted the number of intact sperm in the four 1mm-by-1mm subdivisions at the corners of the counting grids. We averaged the counts across the four corner subdivisions on each counting grids and then multiplied the mean of both counting grids by the dilution factor (2000) to estimate the total sperm in the bursa copulatrix. The bursa copulatrix was not properly preserved in 18 out of the 158 mated females in 2021, and one sperm transfer trial occurred at a mating temperature (*T*_mate_) above the 36°C heat stress limit (Leith *et al.* 2024), resulting in 139 sperm transfer samples included in our analysis.

*Quantifying egg laying.* We tested for carryover effects of developmental temperatures on female fecundity using the mated females from the 2022 mating trials. We placed the females individually on new netted host plants and measured the likelihood of oviposition and the number of eggs laid. Netted plants for the fecundity trials were standardized at ~0.5m tall. We housed the fecundity plants outdoors next to the rearing greenhouse and allowed the females to oviposit into the stems of the plants until the females naturally senesced in the first fall frost. We clipped all stems that had visible egg masses, which are easily identified by a bright white waxy covering excreted by the female following oviposition. We then counted the number of eggs laid by each female after cutting away the top layer of bark on each egg mass with a surgical scalpel (Fowler-Finn *et al.* 2018). Out of the 65 mated females in 2022, nine of the netted fecundity plants tipped over while outdoors (preventing oviposition) and the juveniles hatched early on one plant (preventing egg counts). We therefore included 56 trials in the analysis for egg laying likelihood and 52 trials in the analysis for total eggs laid.

*Statistical analyses characterizing carryover effects on multiple stages of reproduction.* To characterize the types of carryover effects shaping performance at each stage of reproduction, we tested for specific patterns of reproductive success across different combinations of developmental temperatures (*T*_dev_) and adult mating temperatures (*T*_mate_). Silver spoon effects are supported if reproductive performance is greater at all mating temperatures in one female and/or male *T*_dev_ treatment. Silver spoon effects can be sex specific, with effects of female and male *T*_dev_ that differ in magnitude and/or sign. Predictive adaptive plasticity is supported by significant interactions between *T*_dev_ and *T*_mate_, where performance is highest when relative temperatures match across life stages. Predictive adaptive plasticity can also be sex specific. Finally, carryover effects that lead to assortative reproduction by developmental environment are supported by significant interactions between female and male *T*_dev_, with reduced performance when males and females are from different *T*_dev_ treatments. Assortative reproduction carryover effects can be asymmetric, for example, if performance decreases when males from the cold *T*_dev_ treatment are paired with females from the warm *T*_dev_ treatment, but not vice versa. Importantly, these different types of carryover effects are not mutually exclusive and may act simultaneously to shape performance across thermal conditions and reproductive stages.

We characterized carryover effects at each stage of reproduction using a series of generalized linear models (*stats*, R v4.2.0; R Core Team 2022) and visualized all results with *ggplot2* (Wickham 2016). For mating likelihood and egg-laying likelihood, we used binomial generalized linear models. For mating latency, mating duration, sperm transferred, and total eggs laid, we used a natural log-transformation and a gaussian variance distribution. We also divided sperm transfer by 100 and then added 1 before log-transforming to improve the normality of residuals. Models for data collected in 2021 (mating likelihood, mating duration, and sperm transfer) included effects of female developmental temperature (*T*_dev_), male *T*_dev_, adult mating temperature (*T*_mate_), and all their two- and three-way interactions. We also included quadratic *T*_mate_ and all of its two- and three-way interactions with female and male *T*_dev_, since previous work shows that *E. binotata* mating activity is highest at intermediate temperatures (Leith *et al.* 2020; Macchiano *et al.* 2023). Models for data collected in 2022 (mating likelihood, mating duration, egg laying likelihood, and number of eggs laid) included main effects of *T*_dev_, *T*_mate_, quadratic *T*_mate_, and two-way interactions between all *T*_dev_ and *T*_mate_ terms. We treated *T*_dev_ as a two-level factor and *T*_mate_ as a continuous variable in all models. We initially included male and female rearing plant as random effects (*lme4*; Bates *et al.* 2015) but removed them from the final models because they produced singular fits and/or convergence issues. We assessed the significance of each effect using likelihood ratio tests and removed all nonsignificant interactions and quadratic terms from the global models to avoid overparameterization (Table S2).

*Measuring body size and genitalia.* For pronotum and face length, we placed the preserved individuals on their right side into a small weigh boat filled with sand. For all other traits, we removed the relevant body segment (right femur 2, ovipositor, aedeagus, and paired styles) and placed them into the weigh boat individually. We then captured two photographs of the intact body and each body segment, lifting and re-positioning each body segment in between photographs. We measured all traits twice for each photograph using the LAS X software (Leica Microsystems Inc.), averaged the measurements within photographs, and then averaged the means across photographs for all traits.

*Testing effects of developmental temperature on the scaling of body size and genitalia.* We performed log-log regressions to test for effects of developmental temperature on the scaling relationships between body size and genitalia. We used pronotum length as a proxy for body size following methods from previous studies in *E. binotata* (Rodríguez & Al-Wathiqui 2011). After natural log-transforming pronotum length and the five female and male genitalia traits (ovipositor trait 1, ovipositor trait 2, aedeagus trait 1, aedeagus trait 2, and aedeagus trait 3), we fit linear mixed effects models with each genitalia trait as the response variables. We included fixed effects of developmental temperature (*T*_dev_), ln(pronotum length), and their interaction. We specified an offset slope of 1 for ln(pronotum length) to test the null hypothesis that the scaling of each genitalia trait with pronotum length was isometric. Rearing plant replicate was included as a random effect in all models. We determined significance using F tests and the Kenward-Roger degrees of freedom approximation.

*Predictions tested to link carryover effects to selection on reproductive morphology.* If carryover effects shape selection on morphological traits, then an individual’s developmental environment and/or the developmental environment of their mates should influence if and how morphological traits affect reproductive performance. For instance, silver spoon effects may enable more individuals to express high-performance morphological phenotypes when they experience optimal developmental conditions—ultimately exposing those morphological traits to selection. At the same time, carryover effects that promote assortative reproduction by developmental environment could strengthen selection on reproductive morphology, since males and females from different developmental environments may express mismatched morphological traits. In this case, selection should favor individuals with phenotypes that reduce these morphological mismatches (e.g., especially small or especially large trait values). To test these predictions, we constructed separate generalized linear models for data in each of the four *T*_dev_ treatment combinations. Mating likelihood and sperm transferred were fit as the response variables, with effects of *T*_mate_, the male and female morphological traits that showed plasticity in response to *T*_dev_. We did not include effects of female ovipositor 1 and female face length in the same models due to collinearity issues. We therefore included effects of *T*_mate_, male aedeagus 3, one of the two female traits, and their interaction as predictor variables, but removed the interaction from the final models due to frequent convergence issues. Mating likelihood models also often failed to converge when both the male and female trait were included simultaneously, and we therefore only included one morphological trait effect at a time in the mating likelihood models. From the final models, we compared the magnitude and direction of the standardized parameter estimates of each trait effect among *T*_dev_ treatments.

**Supporting information tables**

**Table S1.** Sample size (number of male-female pairs) for each developmental temperature (*T*_dev_) and adult mating temperature (*T*_mate_) treatment combination used in the 2021 and 2022 mating trials.

| **Year** | **Developmental temperature (*T*_dev_)** | **Target mating temperature (*T*_mate_)** | ***n*** |
| --- | --- | --- | --- |
| 2021 | 21°C - 21°C  Female - Male | 23°C | 33 |
|  |  | 28°C | 29 |
|  |  | 33°C | 29 |
|  | 21°C - 26°C  Female - Male | 23°C | 13 |
|  |  | 28°C | 13 |
|  |  | 33°C | 14 |
|  | 26°C - 21°C  Female - Male | 23°C | 11 |
|  |  | 28°C | 12 |
|  |  | 33°C | 12 |
|  | 26°C - 26°C  Female - Male | 23°C | 29 |
|  |  | 28°C | 29 |
|  |  | 33°C | 29 |
| 2022 | 21°C - 21°C  Female - Male | 23°C | 28 |
|  |  | 28°C | 28 |
|  |  | 33°C | 28 |
|  | 26°C - 26°C  Female - Male | 23°C | 29 |
|  |  | 28°C | 28 |
|  |  | 33°C | 28 |

**Table S2.** Results from global generalized linear models testing the effects of female and male developmental temperature (*T*_dev_) and adult mating temperature (*T*_mate_) on each stage of reproductive success. Significant parameters are indicated in bold. Interactions and quadratic effects with *P* > 0.05 were removed from the final models.

| **Year** | **Response** | **Term** | $\boldsymbol{LR}\boldsymbol{\chi}_{\boldsymbol{1}}^{\boldsymbol{2}}$ | **df** | **P** |
| --- | --- | --- | --- | --- | --- |
| 2021 | Mating likelihood | Female developmental temperature (*T*_dev_) | **12.13** | **1** | **0.0005** |
|  |  | Male developmental temperature (*T*_dev_) | **3.91** | **1** | **0.0480** |
|  |  | Mating temperature (*T*_mate_) | **12.94** | **1** | **0.0003** |
|  |  | Quadratic *T*_mate_ | 0.72 | 1 | 0.3958 |
|  |  | Female *T*_dev_ x Male *T*_dev_ | **7.11** | **1** | **0.0077** |
|  |  | Female *T*_dev_ x *T*_mate_ | 0.01 | 1 | 0.9087 |
|  |  | Male *T*_dev_ x *T*_mate_ | 0.16 | 1 | 0.6866 |
|  |  | Female *T*_dev_ x Quadratic *T*_mate_ | 0.36 | 1 | 0.5504 |
|  |  | Male *T*_dev_ x Quadratic *T*_mate_ | 0.06 | 1 | 0.8007 |
|  |  | Female *T*_dev_ x Male *T*_dev_ x *T*_mate_ | 0.51 | 1 | 0.4740 |
|  |  | Female *T*_dev_ x Male *T*_dev_ x Quadratic *T*_mate_ | 0.28 | 1 | 0.5978 |
| 2021 | Mating latency | Female *T*_dev_ | 0.10 | 1 | 0.7517 |
|  |  | Male *T*_dev_ | 0.05 | 1 | 0.8212 |
|  |  | *T*_mate_ | 2.38 | 1 | 0.1233 |
|  |  | Quadratic *T*_mate_ | 1.49 | 1 | 0.2227 |
|  |  | Female *T*_dev_ x Male *T*_dev_ | 0.73 | 1 | 0.3919 |
|  |  | Female *T*_dev_ x *T*_mate_ | 2.86 | 1 | 0.0906 |
|  |  | Male *T*_dev_ x *T*_mate_ | 0.08 | 1 | 0.7780 |
|  |  | Female *T*_dev_ x Quadratic *T*_mate_ | 1.27 | 1 | 0.2605 |
|  |  | Male *T*_dev_ x Quadratic *T*_mate_ | 0.46 | 1 | 0.4991 |
|  |  | Female *T*_dev_ x Male *T*_dev_ x *T*_mate_ | 0.27 | 1 | 0.6063 |
|  |  | Female *T*_dev_ x Male *T*_dev_ x Quadratic *T*_mate_ | 0.27 | 1 | 0.6017 |
| 2021 | Mating duration | Female *T*_dev_ | 0.74 | 1 | 0.3906 |
|  |  | Male *T*_dev_ | 0.06 | 1 | 0.8097 |
|  |  | *T*_mate_ | **73.00** | **1** | **<0.0001** |
|  |  | Quadratic *T*_mate_ | 0.19 | 1 | 0.6622 |
|  |  | Female *T*_dev_ x Male *T*_dev_ | 1.70 | 1 | 0.1926 |
|  |  | Female *T*_dev_ x *T*_mate_ | 0.01 | 1 | 0.9161 |
|  |  | Male *T*_dev_ x *T*_mate_ | 0.12 | 1 | 0.7338 |
|  |  | Female *T*_dev_ x Quadratic *T*_mate_ | 2.23 | 1 | 0.1355 |
|  |  | Male *T*_dev_ x Quadratic *T*_mate_ | 3.43 | 1 | 0.0641 |
|  |  | Female *T*_dev_ x Male *T*_dev_ x *T*_mate_ | 0.03 | 1 | 0.8679 |
|  |  | Female *T*_dev_ x Male *T*_dev_ x Quadratic *T*_mate_ | 0.09 | 1 | 0.7585 |
| 2021 | Sperm transferred | Female *T*_dev_ | **12.91** | **1** | **0.0003** |
|  |  | Male *T*_dev_ | **5.25** | **1** | **0.0220** |
|  |  | *T*_mate_ | 0.41 | 1 | 0.5195 |
|  |  | Quadratic *T*_mate_ | 0.25 | 1 | 0.6191 |
|  |  | Female *T*_dev_ x Male *T*_dev_ | 1.61 | 1 | 0.2046 |
|  |  | Female *T*_dev_ x *T*_mate_ | 0.20 | 1 | 0.6563 |
|  |  | Male *T*_dev_ x *T*_mate_ | 0.61 | 1 | 0.4359 |
|  |  | Female *T*_dev_ x Quadratic *T*_mate_ | 3.35 | 1 | 0.0672 |
|  |  | Male *T*_dev_ x Quadratic *T*_mate_ | 0.30 | 1 | 0.5819 |
|  |  | Female *T*_dev_ x Male *T*_dev_ x *T*_mate_ | 0.20 | 1 | 0.6532 |
|  |  | Female *T*_dev_ x Male *T*_dev_ x Quadratic *T*_mate_ | 0.02 | 1 | 0.8947 |
| 2022 | Mating likelihood | *T*_dev_ | 2.23 | 1 | 0.1356 |
|  |  | *T*_mate_ | **3.96** | **1** | **0.0467** |
|  |  | Quadratic *T*_mate_ | **5.70** | **1** | **0.0169** |
|  |  | *T*_dev_ x *T*_mate_ | 0.30 | 1 | 0.5824 |
|  |  | *T*_dev_ x Quadratic *T*_mate_ | 0.99 | 1 | 0.3186 |
| 2022 | Mating latency | *T*_dev_ | 0.11 | 1 | 0.7390 |
|  |  | *T*_mate_ | 0.05 | 1 | 0.8232 |
|  |  | Quadratic *T*_mate_ | 0.00 | 1 | 0.9678 |
|  |  | *T*_dev_ x *T*_mate_ | 2.25 | 1 | 0.1337 |
|  |  | *T*_dev_ x Quadratic *T*_mate_ | 0.03 | 1 | 0.8573 |
| 2022 | Mating duration | *T*_dev_ | 0.42 | 1 | 0.5186 |
|  |  | *T*_mate_ | **50.48** | **1** | **<0.0001** |
|  |  | Quadratic *T*_mate_ | 2.99 | 1 | 0.0837 |
|  |  | *T*_dev_ x *T*_mate_ | 2.24 | 1 | 0.1344 |
|  |  | *T*_dev_ x Quadratic *T*_mate_ | 0.53 | 1 | 0.4650 |
| 2022 | Egg laying likelihood | *T*_dev_ | **6.18** | **1** | **0.0129** |
|  |  | *T*_mate_ | 0.05 | 1 | 0.8294 |
|  |  | Quadratic *T*_mate_ | 1.26 | 1 | 0.2612 |
|  |  | *T*_dev_ x *T*_mate_ | 0.00 | 1 | 1.0000 |
|  |  | *T*_dev_ x Quadratic *T*_mate_ | 0.00 | 1 | 0.9999 |
| 2022 | Eggs laid | *T*_dev_ | 0.19 | 1 | 0.6649 |
|  |  | *T*_mate_ | 0.00 | 1 | 0.9451 |
|  |  | Quadratic *T*_mate_ | 1.98 | 1 | 0.1593 |
|  |  | *T*_dev_ x *T*_mate_ | 0.00 | 1 | 0.9690 |
|  |  | *T*_dev_ x Quadratic *T*_mate_ | 0.04 | 1 | 0.8397 |

**Table S3.** Results from generalized linear models testing the effects of female and male developmental temperature (*T*_dev_) and adult mating temperature (*T*_mate_) on each stage of reproductive success. The 95% confidence interval for the *T*_dev_ parameter estimate (*β*) could not be calculated for egg-laying likelihood because all mated females laid eggs in the 26°C *T*_dev_ treatment (indicated by dashes). Significant parameters are indicated in bold. Interactions and quadratic effects with *P* > 0.05 were removed from the final models and are therefore not shown. We retained the non-significant quadratic *T*_mate_ effect in the mating likelihood analysis so that the fitted *P*_mate_ curves across *T*_mate_ were comparable between years.

| **Year** | **Response** | **Term** | $\boldsymbol{LR}\boldsymbol{\chi}_{\boldsymbol{1}}^{\boldsymbol{2}}$ | **df** | **P** | ***T*_dev_ *β*** | **Low CI 95%** | **High CI 95%** |
| --- | --- | --- | --- | --- | --- | --- | --- | --- |
| 2021 | Mating likelihood | Female developmental temperature (*T*_dev_) | 12.22 | 1 | **0.0005** | **0.18** | **-0.65** | **1.04** |
|  |  | Male developmental temperature (*T*_dev_) | 3.85 | 1 | **0.0499** | **-1.30** | **-2.13** | **-0.50** |
|  |  | Mating temperature (*T*_mate_) | 13.55 | 1 | **0.0002** | **-0.51** | **-0.80** | **-0.24** |
|  |  | Female *T*_dev_ x Male *T*_dev_ | 7.09 | 1 | **0.0078** | **1.61** | **0.43** | **2.81** |
|  | Sperm transferred | Female *T*_dev_ | 12.71 | 1 | **0.0004** | **-1.18** | **-1.83** | **-0.53** |
|  |  | Male *T*_dev_ | 5.37 | 1 | **0.0205** | **0.77** | **0.12** | **1.43** |
|  |  | *T*_mate_ | 0.96 | 1 | 0.3273 | 0.04 | -0.04 | 0.11 |
| 2022 | Mating likelihood | *T*_dev_ | 2.23 | 1 | 0.1356 | 0.49 | -0.15 | 1.13 |
|  |  | *T*_mate_ | 3.58 | 1 | 0.0584 | -0.32 | -0.67 | 0.01 |
|  |  | Quadratic *T*_mate_ | 5.59 | 1 | **0.0180** | **-0.52** | **-0.97** | **-0.09** |
|  | Egg laying likelihood | *T*_dev_ | 6.16 | 1 | **0.0131** | **18.8** | **-** | **-** |
|  |  | *T*_mate_ | 0.00 | 1 | 0.9475 | 0.04 | -1.18 | 1.26 |
|  | Eggs laid | *T*_dev_ | 0.22 | 1 | 0.6426 | 0.13 | -0.43 | 0.69 |
|  |  | *T*_mate_ | 0.14 | 1 | 0.7081 | 0.06 | -0.24 | 0.35 |

**Table S4.** Results from generalized linear models testing the effects of female and male developmental temperature (*T*_dev_) and adult mating temperature (*T*_mate_) on mating latency and duration. Significant parameters are indicated in bold. Interactions with *P* > 0.05 were removed from the final models and are therefore not shown.

| **Year** | **Response** | **Term** | $\boldsymbol{LR}\boldsymbol{\chi}_{\boldsymbol{1}}^{\boldsymbol{2}}$ | **df** | **P** | ***T*_dev_ *β*** | **Low CI 95%** | **High CI 95%** |
| --- | --- | --- | --- | --- | --- | --- | --- | --- |
| 2021 | Mating latency | Female developmental temperature (*T*_dev_) | 0.08 | 1 | 0.7751 | -0.06 | -0.44 | 0.33 |
|  |  | Male developmental temperature (*T*_dev_) | 0.07 | 1 | 0.7924 | 0.05 | -0.33 | 0.43 |
|  |  | Mating temperature (*T*_mate_) | 2.01 | 1 | 0.1563 | 0.12 | -0.05 | 0.29 |
|  |  | Quadratic *T*_mate_ | 0.99 | 1 | 0.3200 | 0.11 | -0.11 | 0.33 |
|  | Mating duration | Female *T*_dev_ | 0.76 | 1 | 0.3830 | -0.14 | -0.46 | 0.18 |
|  |  | Male *T*_dev_ | 0.07 | 1 | 0.7927 | 0.04 | -0.28 | 0.36 |
|  |  | *T*_mate_ | **75.59** | **1** | **<0.0001** | **-0.61** | **-0.75** | **-0.47** |
|  |  | Quadratic *T*_mate_ | 0.19 | 1 | 0.6627 | -0.04 | -0.22 | 0.14 |
| 2022 | Mating latency | *T*_dev_ | 0.11 | 1 | 0.7398 | 0.09 | -0.42 | 0.59 |
|  |  | *T*_mate_ | 0.03 | 1 | 0.8578 | -0.02 | -0.28 | 0.23 |
|  |  | Quadratic *T*_mate_ | 0.07 | 1 | 0.7891 | 0.04 | -0.25 | 0.32 |
|  | Mating duration | *T*_dev_ | 0.41 | 1 | 0.5205 | -0.12 | -0.50 | 0.25 |
|  |  | *T*_mate_ | **49.83** | **1** | **<0.0001** | **-0.69** | **-0.88** | **-0.50** |
|  |  | Quadratic *T*_mate_ | 2.38 | 1 | 0.1225 | 0.17 | -0.04 | 0.38 |

**Table S5.** Results from post hoc contrasts of the estimated marginal means of mating likelihood across each male and female *T*_dev_ combination in the 2021 mating trials. Significant parameters are indicated in bold.

| **Contrast** | ***β*** | **SE** | **Z ratio** | **P** |
| --- | --- | --- | --- | --- |
| 21°C F *T*_dev_ & 21°C M *T*_dev_ - 26°C F *T*_dev_ & 21°C M *T*_dev_ | -0.18 | 0.43 | -0.42 | 0.6710 |
| 21°C F *T*_dev_ & 21°C M *T*_dev_ - 21°C F *T*_dev_ & 26°C M *T*_dev_ | **1.30** | **0.41** | **3.13** | **0.0017** |
| 21°C F *T*_dev_ & 21°C M *T*_dev_ - 26°C F *T*_dev_ & 26°C M *T*_dev_ | -0.50 | 0.33 | -1.50 | 0.1342 |
| 26°C F *T*_dev_ & 21°C M *T*_dev_ - 21°C F *T*_dev_ & 26°C M *T*_dev_ | **1.48** | **0.51** | **2.92** | **0.0035** |
| 26°C F *T*_dev_ & 21°C M *T*_dev_ - 26°C F *T*_dev_ & 26°C M *T*_dev_ | -0.32 | 0.44 | -0.72 | 0.4711 |
| 21°C F *T*_dev_ & 26°C M *T*_dev_ - 26°C F *T*_dev_ & 26°C M *T*_dev_ | **-1.80** | **0.43** | **-4.19** | **<0.0001** |

**Table S6.** Effects of rearing treatment on the allometric scaling of male and female morphological traits. We specified an offset slope of 1 for ln(Pronotum length) to test the null hypothesis that the scaling of each genitalia trait with pronotum length was isometric. Rearing plant was included as a random effect in all models. Results were obtained from linear mixed-effects models using F tests and the Kenward-Roger degrees of freedom approximation. Significant parameters are indicated in bold.

| **Response** | **Variable** | **F** | **df** | **P** |
| --- | --- | --- | --- | --- |
| Female  ln(Ovipositor trait 1) | *T*_dev_ | 4.83 | 1, 13.16 | **0.0464** |
|  | ln(Pronotum length) | 59.62 | 1, 119.28 | **<0.0001** |
|  | *T*_dev_ x ln(Pronotum length) | 0.05 | 1, 123.21 | 0.8159 |
| Female  ln(Ovipositor trait 2) | *T*_dev_ | 0.05 | 1, 13.47 | 0.8304 |
|  | ln(Pronotum length) | 49.00 | 1, 126.47 | **<0.0001** |
|  | *T*_dev_ x ln(Pronotum length) | 0.14 | 1, 130.57 | 0.7085 |
| Male  ln(Aedeagus trait 1) | *T*_dev_ | 1.11 | 1, 12.31 | 0.3127 |
|  | ln(Pronotum length) | 220.58 | 1, 108.61 | **<0.0001** |
|  | *T*_dev_ x ln(Pronotum length) | 0.33 | 1, 107.96 | 0.5642 |
| Male  ln(Aedeagus trait 2) | *T*_dev_ | 0.76 | 1, 12.23 | 0.4013 |
|  | ln(Pronotum length) | 236.69 | 1, 104.54 | **<0.0001** |
|  | *T*_dev_ x ln(Pronotum length) | 0.57 | 1, 104.38 | 0.4525 |
| Male  ln(Aedeagus trait 3) | *T*_dev_ | 7.65 | 1, 12.96 | **0.0161** |
|  | ln(Pronotum length) | 65.52 | 1, 147.72 | **<0.0001** |
|  | *T*_dev_ x ln(Pronotum length) | 0.03 | 1, 140.57 | 0.8672 |
| Male  ln(Style trait 1) | *T*_dev_ | 4.05 | 1, 12.39 | 0.0664 |
|  | ln(Pronotum length) | 101.20 | 1, 103.18 | **<0.0001** |
|  | *T*_dev_ x ln(Pronotum length) | 0.01 | 1, 108.64 | 0.9302 |
| Male  ln(Style trait 2) | *T*_dev_ | 4.00 | 1, 12.39 | 0.0678 |
|  | ln(Pronotum length) | 41.97 | 1, 103.62 | **<0.0001** |
|  | *T*_dev_ x ln(Pronotum length) | 0.25 | 1, 108.73 | 0.6309 |

**Table S7.** Effects of developmental temperature (*T*_dev_) on male and female morphological traits. Rearing plant was included as a random effect in all models. The number of individuals measured in each treatment are listed under *n*. Results were obtained from linear mixed-effects models using F tests and the Kenward-Roger degrees of freedom approximation. Significant parameters are indicated in bold.

| **Response** | ***n* 21°C** | ***n* 26°C** | **F** | **df** | **P** | ***T*_dev_ *β*** | **Low CI 95%** | **High CI 95%** |
| --- | --- | --- | --- | --- | --- | --- | --- | --- |
| Female pronotum length | 112 | 89 | 0.06 | 1, 14.02 | 0.8077 | 0.07 | -0.47 | 0.61 |
| Female face length | 119 | 91 | 4.61 | 1, 14.06 | **0.0498** | **0.49** | **0.05** | **0.94** |
| Female femur length | 124 | 101 | 0.70 | 1, 14.05 | 0.4174 | -0.17 | -0.56 | 0.23 |
| Female ovipositor 1 | 110 | 97 | 5.17 | 1, 13.84 | **0.0395** | **0.31** | **0.04** | **0.58** |
| Female ovipositor 2 | 112 | 91 | 0.00 | 1, 14.18 | 0.9683 | 0.01 | -0.27 | 0.28 |
| Male pronotum length | 96 | 104 | 0.07 | 1, 13.98 | 0.7964 | -0.08 | -0.67 | 0.51 |
| Male face length | 104 | 110 | 1.74 | 1, 13.92 | 0.2087 | 0.31 | -0.15 | 0.78 |
| Male femur length | 114 | 115 | 0.01 | 1, 13.96 | 0.9084 | -0.03 | -0.52 | 0.46 |
| Male aedeagus 1 | 108 | 107 | 0.50 | 1, 13.31 | 0.4908 | 0.11 | -0.19 | 0.41 |
| Male aedeagus 2 | 108 | 107 | 0.85 | 1, 13.68 | 0.3722 | -0.18 | -0.55 | 0.19 |
| Male aedeagus 3 | 110 | 108 | 11.75 | 1, 13.49 | **0.0043** | **0.52** | **0.22** | **0.81** |
| Male style 1 | 113 | 100 | 2.07 | 1, 13.69 | 0.1726 | 0.23 | -0.08 | 0.55 |
| Male style 2 | 113 | 102 | 2.80 | 1, 13.44 | 0.1174 | 0.23 | -0.04 | 0.50 |

**Table S8.** Results of generalized linear models testing the effects of female and male morphological traits on mating likelihood across combinations of female and male developmental temperature (*T*_dev_). Mating temperature (*T*_mate_) was included as a covariate in all models. However, the parameter estimates for *T*_mate_ are not reported here for simplicity, as all trait effects on mating likelihood were analyzed in separate models (see Methods for justification). Significant parameters are indicated in bold.

| ***T*_dev_** | **Variable** | ***n*** | **LR** $\boldsymbol{\chi}_{\boldsymbol{1}}^{\boldsymbol{2}}$ | **df** | **P** | ***β*** | **Low CI 95%** | **High CI 95%** |
| --- | --- | --- | --- | --- | --- | --- | --- | --- |
| 21F & 21M | Female face length | 84 | 0.05 | 1 | 0.8317 | 0.05 | -0.41 | 0.52 |
| 21F & 21M | Female ovipositor trait 1 | **74** | **7.71** | **1** | **0.0055** | **-0.79** | **-1.54** | **-0.21** |
| 21F & 21M | Male aedeagus trait 3 | 83 | 3.08 | 1 | 0.0795 | 0.41 | -0.05 | 0.92 |
| 21F & 26M | Female face length | 35 | 2.32 | 1 | 0.1276 | 0.67 | -0.19 | 1.68 |
| 21F & 26M | Female ovipositor trait 1 | 36 | 3.48 | 1 | 0.0621 | -0.74 | -1.70 | 0.04 |
| 21F & 26M | Male aedeagus trait 3 | **35** | **3.86** | **1** | **0.0494** | **-0.91** | **-2.03** | **0.00** |
| 26F & 21M | Female face length | **19** | **7.71** | **1** | **0.0055** | **-2.36** | **-5.71** | **-0.50** |
| 26F & 21M | Female ovipositor trait 1 | 20 | 0.01 | 1 | 0.9197 | -0.05 | -1.05 | 1.03 |
| 26F & 21M | Male aedeagus trait 3 | **27** | **11.39** | **1** | **0.0007** | **1.97** | **0.71** | **3.95** |
| 26F & 26M | Female face length | 72 | 0.10 | 1 | 0.7536 | 0.09 | -0.49 | 0.68 |
| 26F & 26M | Female ovipositor trait 1 | 77 | 1.32 | 1 | 0.2513 | -0.35 | -1.03 | 0.23 |
| 26F & 26M | Male aedeagus trait 3 | 73 | 0.14 | 1 | 0.7038 | -0.11 | -0.69 | 0.43 |

**Table S9.** Results of generalized linear models testing the effects of female and male morphological traits on sperm transfer across combinations of female and male developmental temperature (*T*_dev_). Significant parameters are indicated in bold.

| ***T*_dev_** | ***n*** | **Variable** | **LR** $\boldsymbol{\chi}_{\boldsymbol{1}}^{\boldsymbol{2}}$ | **df** | **P** | ***β*** | **Low CI 95%** | **High CI 95%** |
| --- | --- | --- | --- | --- | --- | --- | --- | --- |
| 21F & 21M | 48 | Female face length | 0.13 | 1 | 0.7230 | -0.06 | -0.41 | 0.28 |
|  |  | Male aedeagus trait 3 | 0.00 | 1 | 0.9863 | 0.00 | -0.31 | 0.31 |
|  |  | Mating temperature (*T*_mate_) | 0.77 | 1 | 0.3816 | 0.15 | -0.18 | 0.48 |
| 21F & 26M | 9 | Female face length | 0.27 | 1 | 0.6021 | 0.32 | -0.87 | 1.51 |
|  |  | Male aedeagus trait 3 | 1.64 | 1 | 0.1999 | -0.45 | -1.14 | 0.24 |
|  |  | Mating temperature (*T*_mate_) | 0.18 | 1 | 0.6721 | -0.21 | -1.19 | 0.77 |
| 26F & 21M | 10 | Female face length | **8.03** | **1** | **0.0046** | **-1.83** | **-3.09** | **-0.56** |
|  |  | Male aedeagus trait 3 | 2.24 | 1 | 0.1341 | -0.93 | -2.14 | 0.29 |
|  |  | Mating temperature (*T*_mate_) | 3.18 | 1 | 0.0744 | 0.51 | -0.05 | 1.07 |
| 26F & 26M | 37 | Female face length | 0.04 | 1 | 0.8327 | 0.03 | -0.29 | 0.36 |
|  |  | Male aedeagus trait 3 | **4.72** | **1** | **0.0298** | **0.33** | **0.03** | **0.63** |
|  |  | Mating temperature (*T*_mate_) | 0.42 | 1 | 0.5184 | 0.12 | -0.24 | 0.47 |
| 21F & 21M | 38 | Female ovipositor trait 1 | 2.39 | 1 | 0.1218 | -0.19 | -0.44 | 0.05 |
|  |  | Male aedeagus trait 3 | 0.07 | 1 | 0.7967 | 0.05 | -0.31 | 0.40 |
|  |  | Mating temperature (*T*_mate_) | 1.36 | 1 | 0.2436 | 0.21 | -0.14 | 0.55 |
| 21F & 26M | 10 | Female ovipositor trait 1 | 0.26 | 1 | 0.6126 | -0.16 | -0.77 | 0.45 |
|  |  | Male aedeagus trait 3 | 2.17 | 1 | 0.1407 | -0.51 | -1.18 | 0.17 |
|  |  | Mating temperature (*T*_mate_) | 0.02 | 1 | 0.8849 | -0.06 | -0.86 | 0.74 |
| 26F & 21M | 11 | Female ovipositor trait 1 | 0.51 | 1 | 0.4745 | -0.23 | -0.86 | 0.40 |
|  |  | Male aedeagus trait 3 | 0.26 | 1 | 0.6133 | -0.41 | -1.99 | 1.17 |
|  |  | Mating temperature (*T*_mate_) | 3.06 | 1 | 0.0802 | 0.62 | -0.07 | 1.32 |
| 26F & 26M | 40 | Female ovipositor trait 1 | 2.45 | 1 | 0.1174 | -0.20 | -0.46 | 0.05 |
|  |  | Male aedeagus trait 3 | 3.49 | 1 | 0.0618 | 0.26 | -0.01 | 0.53 |
|  |  | Mating temperature (*T*_mate_) | 0.97 | 1 | 0.3255 | 0.15 | -0.15 | 0.45 |

**Supporting information figures**


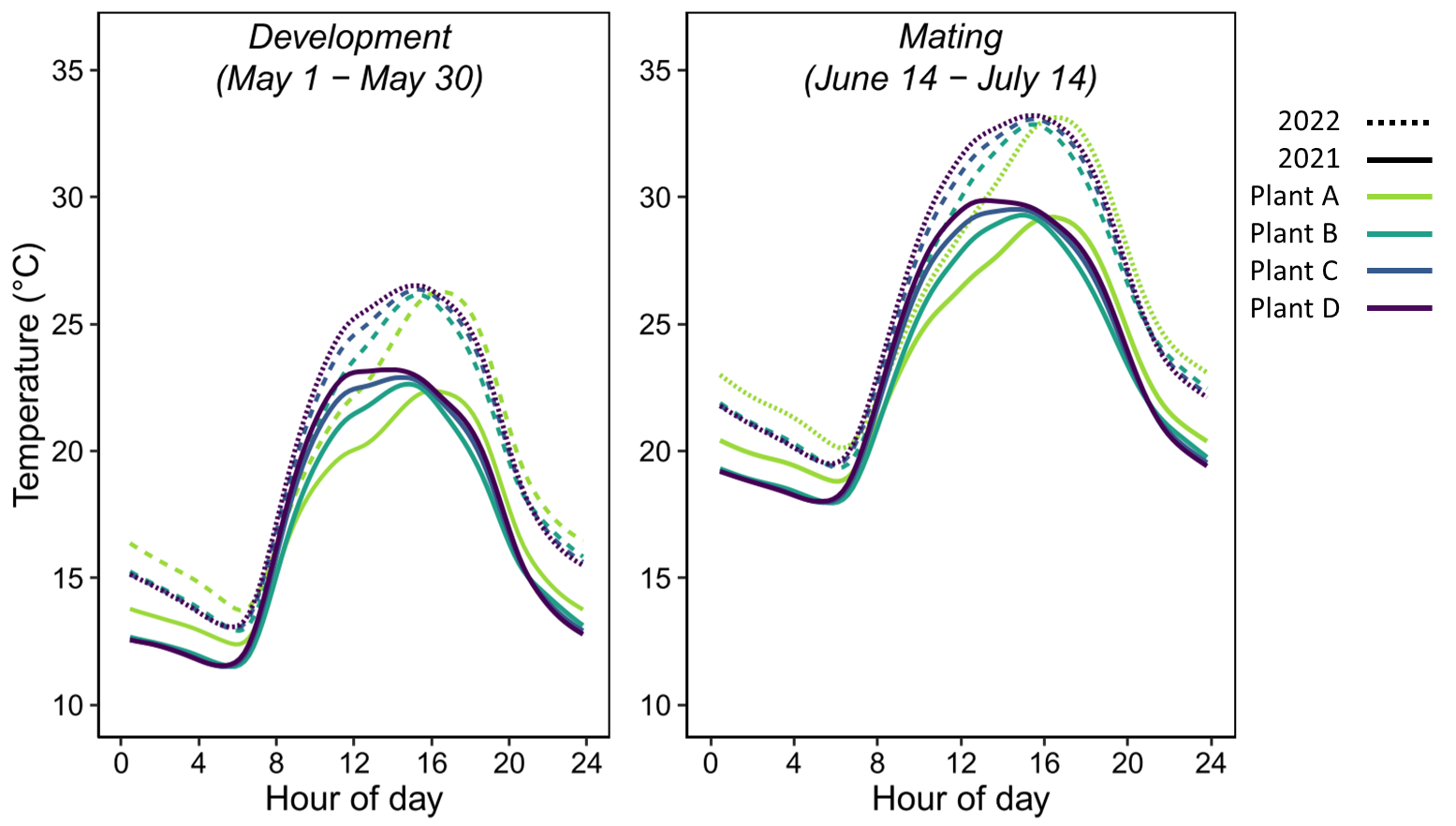


**Figure S1.** Temperature regimes on plants where *E. binotata* were collected in the field. (a) Temperatures regimes measured during the developmental stage (May 1 – May 30). (b) Temperature regimes measured during peak mating season (June 14 – July 14). Curve colors represent temperatures measured on different collection plants. Solid curves represent temperatures measured in 2021, while dashed curves indicate temperatures measured in 2022. Curves were derived from a cyclic generalized additive mixed effects model.


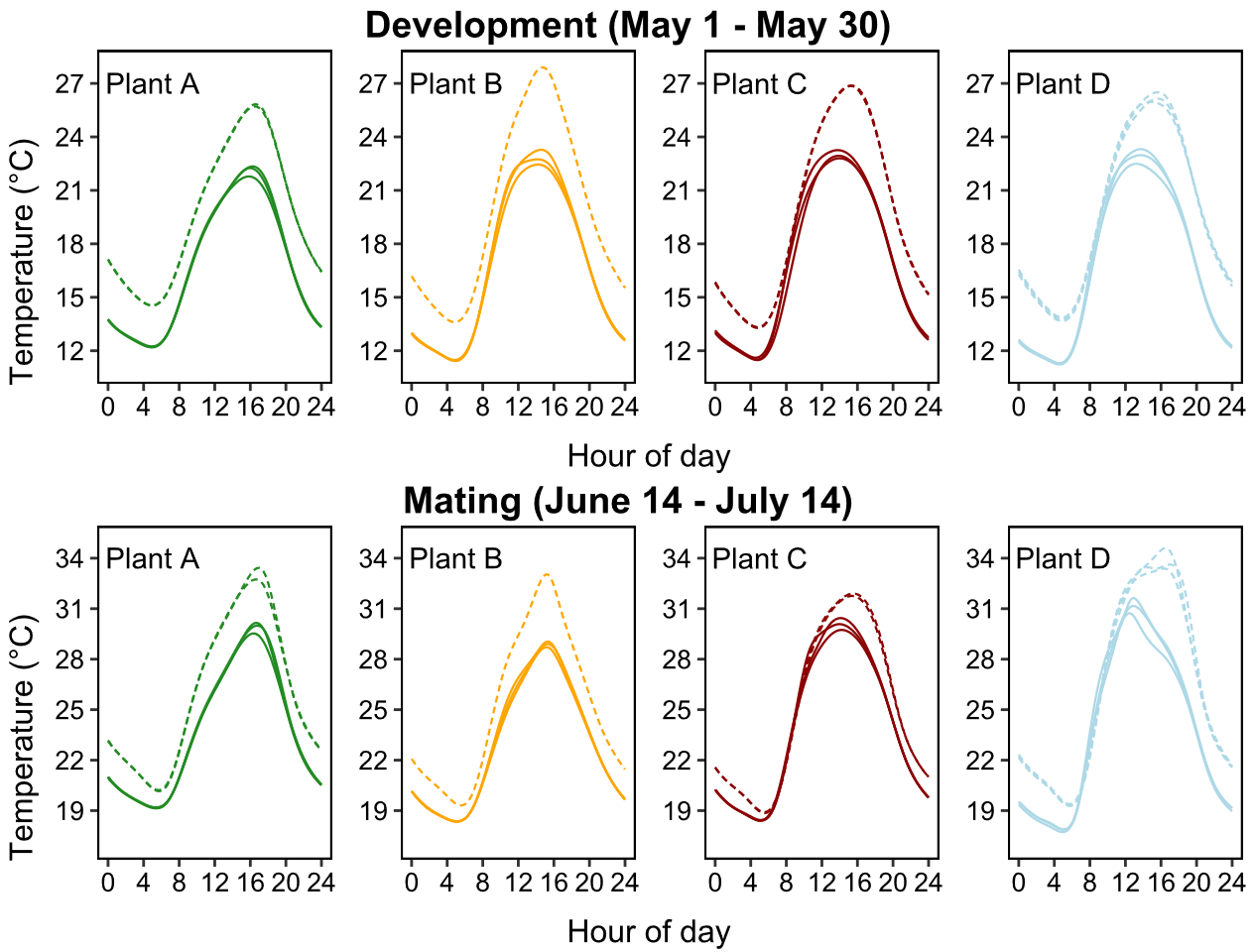


**Figure S2.** Variation in temperature regimes within plants where *E. binotata* were collected in the field. Individual curves represent individual iButton temperature loggers. Curve colors represent temperatures measured on different collection plants. Solid curves represent temperatures measured in 2021, while dashed curves indicate temperatures measured in 2022. The top row show temperature regimes measured during the developmental stage (May 1 – May 30). The bottom row shows temperature regimes measured during peak mating season (June 14 – July 14). Curves were derived from cyclic generalized additive mixed effects models.

**
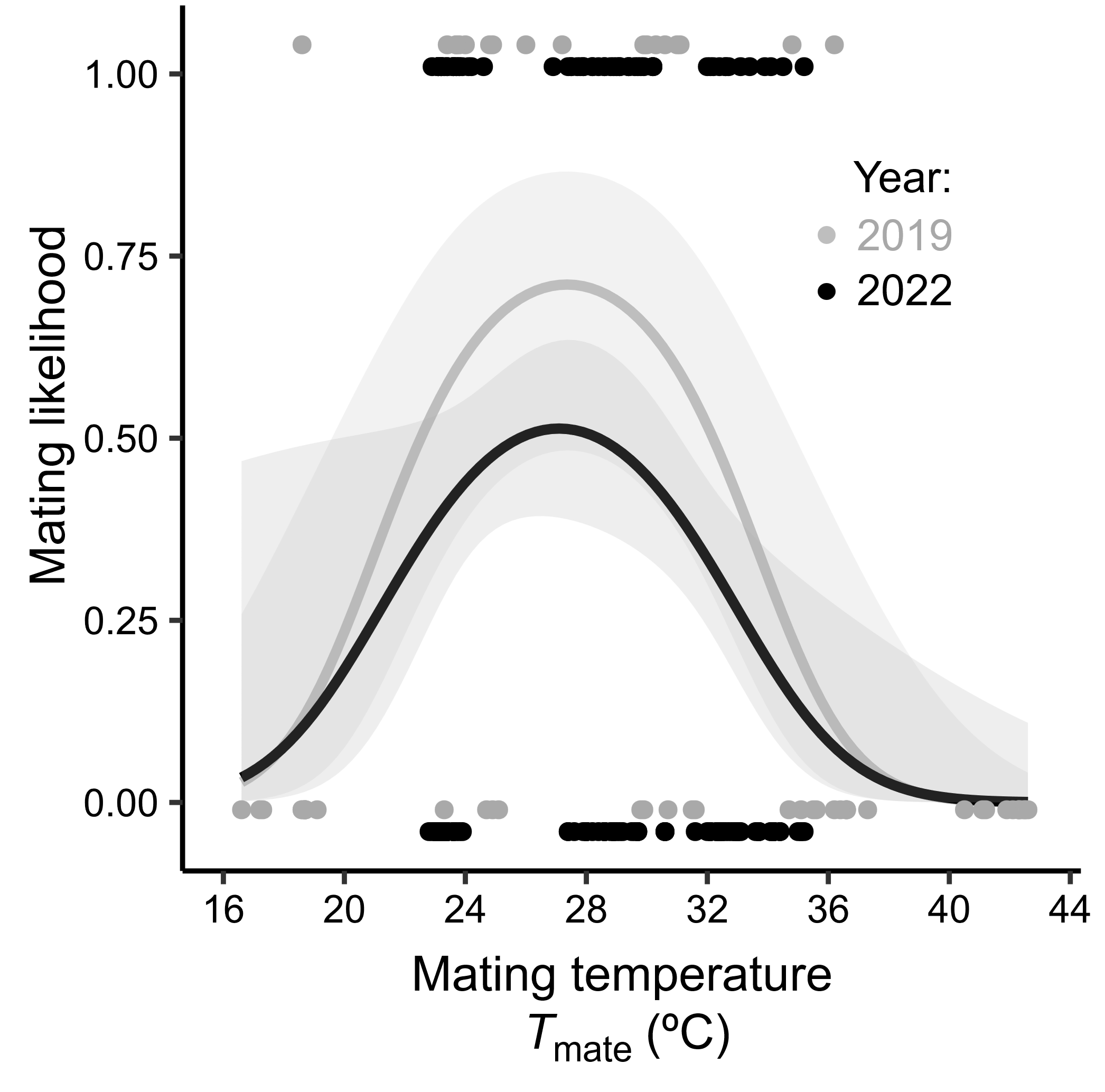
**

**Figure S3.** Mating likelihood in 2022 was highest at intermediate *T*_mate_. reflecting previous results across a broader *T*_mate_ range. The black curve and points in show results from the 2022 mating trials in this study and the grey lines and points show results from an experiment performed in 2019 that tested mating likelihood across a wider range of mating temperatures (Macchiano *et al.* 2023). Lines and bands are estimated fits ± 95% CI from GLMs.

**
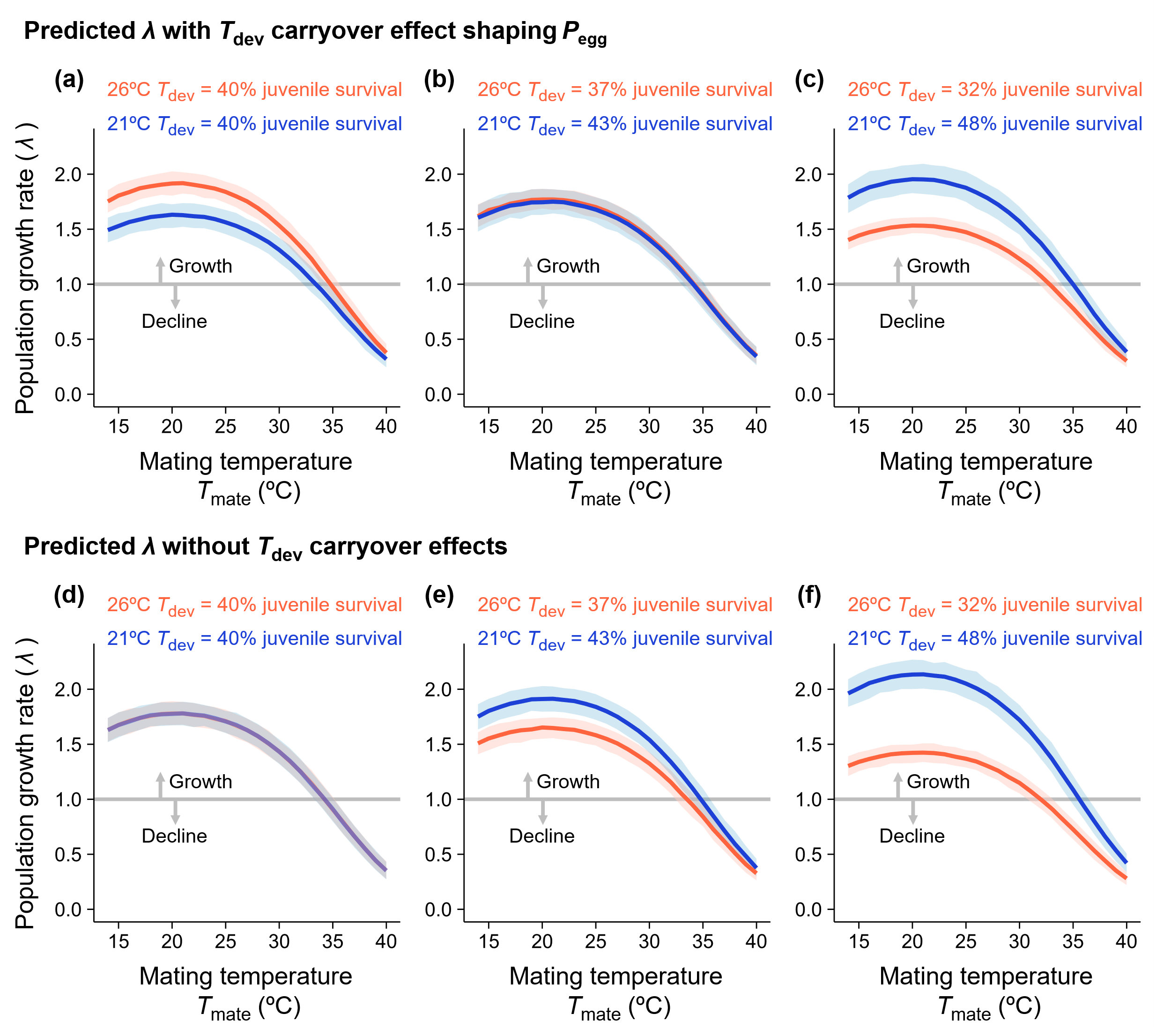
**

**Figure S4.** Simulation model estimating variation in *E. binotata* population growth rate (*λ*) across developmental temperatures (*T*_dev_) and adult mating temperatures (*T*_mate_) where the effect of *T*_mate_ on mating likelihood was parameterized using data from the 2021 mating trials. In (a-c), the probability for mated females to lay eggs depended on *T*_dev_. In (d-f), the probability for mated females to lay eggs was fixed at 0.93. The effect of *T*_dev_ on offspring survival as juveniles varied between no effect (a and d), a moderate effect (b and e), or a severe effect (c and f). Curves and bands show the means and 95% CI for *λ* across 500 simulations. Blue curves indicate 21°C *T*_dev_ and red curves indicate 26°C *T*_dev_.
